# Supplementary material for: Electrophysiological and Behavioral Responses of Virgin Female Bactrocera tryoni to Microbial Volatiles from Enterobacteriaceae
Source: Microorganisms. 2023 Jun 23;11(7):1643. doi: 10.3390/microorganisms11071643 (PMC10385192; doi:10.3390/microorganisms11071643)
Supplement: Supplementary file 1 [file microorganisms-11-01643-s001.zip › microorganisms-2432343-supplementary.pdf]

Supplementary materials:

Table S1. Raw EAG response measurements (mV) from sexually immature female *Bactrocera tryoni* to Tenax® extracts from each bacterium (Control tryptone soya broth (TSB) media; CF: *Citrobacter freundii*; EC: *Enterobacter cloacae*; KO: *Klebsiella oxytoca*; PA: *Enterobacter (sy. Pantoea) agglomerans*).

Run_ID	Replicate	Bacteria	Concentration	Time	Intensity
1	2	KO	(10^-1)	14.69	0.15
2	2	CF	conc	7.66	0.18
3	2	Control	conc	16.69	0.21
4	2	Control	(10^-1)	17.72	0.22
5	2	Control	(10^-2)	18.69	0.25
6	2	EC	conc	10.67	0.3
7	2	PA	(10^-1)	2.17	0.31
8	2	EC	(10^-1)	11.75	0.31
9	2	PA	conc	1.16	0.35
10	2	CF	(10^-2)	9.66	0.4
11	2	EC	(10^-2)	12.67	0.4
12	2	PA	(10^-2)	3.17	0.51
13	2	KO	conc	13.69	0.51
14	2	CF	(10^-1)	8.68	0.6
15	2	KO	(10^-2)	15.7	0.77
16	3	EC	(10^-2)	4.28	0.013
17	3	PA	conc	11.3	0.023
18	3	PA	(10^-2)	13.28	0.029
19	3	KO	(10^-1)	6.28	0.033
20	3	EC	(10^-1)	3.29	0.048
21	3	Control	(10^-1)	9.3	0.05
22	3	PA	(10^-1)	12.28	0.058
23	3	CF	(10^-2)	18.29	0.07
24	3	KO	conc	5.28	0.077
25	3	EC	conc	2.28	0.098
26	3	KO	(10^-2)	7.28	0.12
27	3	Control	(10^-2)	10.28	0.14
28	3	CF	conc	17.3	0.18
29	3	Control	conc	8.3	0.2
30	3	CF	(10^-1)	18.29	0.22
31	4	Control	(10^-2)	16.24	0.15
32	4	KO	(10^-1)	11.74	0.17
33	4	EC	(10^-2)	19.25	0.17
34	4	EC	(10^-1)	18.22	0.21
35	4	KO	(10^-2)	12.74	0.26
36	4	EC	conc	17.21	0.28
37	4	Control	conc	13.92	0.33
38	4	Control	(10^-1)	15.24	0.34

39	4	CF	(10^-1)	5.72	0.38
40	4	KO	conc	10.75	0.39
41	4	PA	(10^-1)	2.73	0.41
42	4	CF	(10^-2)	6.74	0.42
43	4	PA	conc	1.73	0.44
44	4	CF	conc	4.73	0.52
45	4	PA	(10^-2)	3.73	0.59
46	5	Control	(10^-2)	0.77	0.23
47	5	Control	(10^-1)	1.76	0.27
48	5	Control	conc	2.61	0.48
49	5	KO	(10^-2)	3.61	0.59
50	5	KO	(10^-1)	4.6	0.4
51	5	KO	conc	5.61	0.7
52	5	EC	(10^-2)	6.6	0.5
53	5	EC	(10^-1)	7.61	0.52
54	5	EC	conc	8.61	0.24
55	5	PA	(10^-2)	9.6	0.21
56	5	PA	(10^-1)	10.62	0.5
57	5	PA	conc	11.62	0.25
58	5	CF	(10^-2)	12.61	0.29
59	5	CF	(10^-1)	13.62	0.28
60	5	CF	conc	14.62	0.11
61	6	CF	(10^-2)	17.98	0.015
62	6	CF	conc	19.98	0.017
63	6	Control	(10^-1)	12.47	0.019
64	6	CF	(10^-1)	18.97	0.032
65	6	PA	(10^-2)	7.96	0.065
66	6	Control	(10^-2)	11.46	0.071
67	6	PA	conc	9.96	0.092
68	6	PA	(10^-1)	8.97	0.094
69	6	KO	(10^-2)	4.48	0.12
70	6	Control	conc	13.47	0.14
71	6	KO	(10^-1)	5.48	0.15
72	6	EC	(10^-1)	1.98	0.23
73	6	KO	conc	6.45	0.25
74	6	EC	conc	2.97	0.4
75	6	EC	(10^-2)	0.98	0.57
76	7	Control	(10^-2)	8.48	0
77	7	CF	(10^-2)	11.98	0
78	7	CF	(10^-1)	13	0
79	7	PA	(10^-2)	15.47	0.071
80	7	Control	conc	10.47	0.13
81	7	CF	conc	14	0.15
82	7	KO	(10^-1)	2.51	0.16
83	7	Control	(10^-1)	9.47	0.16
84	7	PA	conc	17.44	0.16
85	7	EC	conc	20.95	0.2
86	7	PA	(10^-1)	16.45	0.21

87	7	EC	(10^-2)	19.92	0.24
88	7	EC	(10^-1)	18.93	0.29
89	7	KO	(10^-2)	1.51	0.38
90	7	KO	conc	3.5	0.4

Table S2. Mean relative proportions of microbial volatile organic compounds (MVOCs) identified for each bacterium grown at pH3, pH5, PH7, pH9, and pH12, and collected via 24 h headspace collection with solid phase micro-extraction (SPME) fibre. The MVOCs highlighted in grey were used in dual choice behavioural assays with individual compounds. (CF: *Citrobacter freundii*; EC: *Enterobacter cloacae*; KO: *Klebsiella oxytoca*; PA: *Enterobacter (syn. Pantoea) agglomerans* and tryptone soya broth (TSB) media).*KI: Kovats retention index; **RT: retention time (in min).

Sample_ID	pH	bacteria	Compound_ID	RT	Area	KI
5 mix pH12_SPME	pH12	5 mix	Methane, nitroso-	2.735	32727063	
CF pH5_SPME	pH5	CF	Methane, nitroso-	2.726	15100194	
EC pH7_SPME	pH7	EC	Ethanol, 2-nitro-	2.73	15850047	
TSB pH12_SPME	pH12	TSB	Methane, nitroso-	2.731	33020099	
5 mix pH3_SPME	pH3	5 mix	Cyclopropane-1,2,3-d3-methanol, (1.alpha.,2.beta.,3.beta.)-	2.786	3383294	
5 mix pH7_SPME	pH7	5 mix	Pentane-1,5-d2	2.811	18379121	
5 mix pH9_SPME	pH9	5 mix	Pentane-1,5-d2	2.836	18684857	
Cf_SPMEpH7	pH7	CF	2-Propanamine	2.845	16694357	
EA pH12_SPME	pH12	EA	Methane, nitroso-	2.811	30133248	
EA pH3_SPME	pH3	EA	2-Propanone	2.824	16148634	
EA_SPMEpH7	pH7	EA	2-Propanone	2.811	19129082	
EC pH12_SPME	pH12	EC	Methane, nitroso-	2.781	19887503	
EC pH3_SPME	pH3	EC	Ethanol, 2-nitro-	2.756	8876628	
EC pH9_SPME	pH9	EC	Ethanol, 2-nitro-	2.799	5525906	
KO pH12_SPME	pH12	KO	Methane, nitroso-	2.819	8145016	
PA pH12_SPME	pH12	PA	Methane, nitroso-	2.752	18925042	
PA pH3_SPME really	pH3	PA	Pentane-1,5-d2	2.82	5500912	
CF pH3_SPME	pH3	CF	Pentane-1,5-d2	2.896	2936139	503
CF pH9_SPME	pH9	CF	Pentane-1,5-d2	2.87	5044134	
EA pH5_SPME	pH5	EA	Pentane-1,5-d2	2.867	5949860	
EA pH9_SPME	pH9	EA	2-Propanone	2.896	17807632	503

KO pH5_SPME	pH5	KO	2-Propanone	2.866	16357770	
TSB pH3_SPME	pH3	TSB	Cyclopropane-1,2,3-d3-methanol, (1.alpha.,2.beta.,3.beta.)-	2.879	4487250	
TSB pH9_SPME	pH9	TSB	Pentane-1,5-d2	2.858	4750913	
TSB_SPMEpH7	pH7	TSB	Pentane-1,5-d2	2.913	9225197	507
EA pH12_SPME	pH12	EA	2-Propanone, 1-methoxy-	3.078	5398403	545
EA_SPMEpH7	pH7	EA	2-Propanone	3.065	4243315	542
TSB pH12_SPME	pH12	TSB	Methane, nitroso-	3.086	4676494	547
5 mix pH12_SPME	pH12	5 mix	1,3-Butanediol, (S)-	3.205	10910823	574
EA pH12_SPME	pH12	EA	2-Propanone, 1-methoxy-	3.192	5901320	571
EA_SPMEpH7	pH7	EA	2-Butanone	3.247	8361146	583
TSB pH12_SPME	pH12	TSB	Methane, nitroso-	3.179	5944003	568
5 mix pH3_SPME	pH3	5 mix	2-Butanone	3.331	59181530	601
5 mix pH5_SPME	pH5	5 mix	2-Butanone	3.315	90790810	599
5 mix pH7_SPME	pH7	5 mix	2-Butanone	3.327	91497863	601
5 mix pH9_SPME	pH9	5 mix	2-Butanone	3.319	73436062	600
CF pH3_SPME	pH3	CF	2-Butanone	3.302	9200378	596
CF pH9_SPME	pH9	CF	2-Butanone	3.314	16479775	599
Cf_SPMEpH7	pH7	CF	2-Butanone	3.306	40256436	597
EA pH12_SPME	pH12	EA	3(2H)-Furanone, dihydro-2-methyl-	3.302	2972296	596
EA pH3_SPME	pH3	EA	3(2H)-Furanone, dihydro-2-methyl-	3.26	15402733	586
EA pH5_SPME	pH5	EA	2-Butanone	3.294	2914964	594
EA pH9_SPME	pH9	EA	2-Propanone	3.264	4236828	587
EC pH5_SPME	pH5	EC	2-Butanone	3.302	9266304	596
EC pH7_SPME	pH7	EC	2-Butanone	3.297	16742751	595
KO pH3_SPME	pH3	KO	2-Butanone	3.302	10677521	596
KO pH5_SPME	pH5	KO	2-Butanone	3.31	48534120	598
KO pH9_SPME	pH9	KO	2-Butanone	3.306	14800755	597
KO1_SPMEpH7	pH7	KO	2-Butanone	3.294	42881938	594
TSB pH3_SPME	pH3	TSB	Acetic acid	3.285	26996454	592
TSB pH5_SPME	pH5	TSB	Acetic acid	3.289	66614208	593
5 mix pH12_SPME	pH12	5 mix	2-Butanone	3.446	59125406	609
KO pH12_SPME	pH12	KO	2-Butanone	3.433	8672008	608
EA pH12_SPME	pH12	EA	2-Butanone	3.534	11453915	615

EC pH12_SPME	pH12	EC	2-Butanone	3.496	12284402	612
EC pH7_SPME	pH7	EC	2-Butanone	3.483	28796465	611
EC pH9_SPME	pH9	EC	2-Butanone	3.501	5840659	613
PA pH3_SPME really	pH3	PA	2-BUTANONE, 3-METHYL-	3.627	5795256	622
PA pH12_SPME	pH12	PA	ISOBUTYLALCOHOL	3.666	27231233	624
PA_SPMEpH7	pH7	PA	ISOBUTYLALCOHOL	3.67	7525547	625
KO pH3_SPME	pH3	KO	Butanal, 3-methyl-	4.11	6028875	655
KO pH9_SPME	pH9	KO	Butanal, 3-methyl-	4.109	12670263	655
PA pH9_SPME	pH9	PA	Butanal, 3-methyl-	4.063	5935816	652
PA_SPMEpH7	pH7	PA	ISOBUTYLALCOHOL	4.051	7169840	651
TSB pH3_SPME	pH3	TSB	Butanal, 3-methyl-	4.076	26799616	653
TSB pH5_SPME	pH5	TSB	Butanal, 3-methyl-	4.13	70846116	657
TSB pH9_SPME	pH9	TSB	Butanal, 3-methyl-	4.105	6631084	655
TSB_SPMEpH7	pH7	TSB	Butanal, 3-methyl-	4.126	30421703	656
CF pH3_SPME	pH3	CF	Butanal, 3-methyl-	4.156	31949836	659
CF pH3_SPME	pH3	CF	Butanal, 3-methyl-	4.3	18817777	669
TSB pH3_SPME	pH3	TSB	Butanal, 3-methyl-	4.3	17799299	669
TSB_SPMEpH7	pH7	TSB	Silanediol, dimethyl-	4.308	14767550	669
5 mix pH3_SPME	pH3	5 mix	Butanal, 3-methyl-	4.448	8382755	679
KO pH3_SPME	pH3	KO	Butanal, 3-methyl-	4.444	9571472	679
KO pH9_SPME	pH9	KO	Butanal, 3-methyl-	4.38	9955944	674
PA pH9_SPME	pH9	PA	Butanal, 3-methyl-	4.38	5715966	674
CF pH9_SPME	pH9	CF	Silanediol, dimethyl-	4.541	20972510	686
Cf_SPMEpH7	pH7	CF	Silanediol, dimethyl-	4.528	16730191	685
EA pH12_SPME	pH12	EA	Silanediol, dimethyl-	4.473	13493541	681
EA pH3_SPME	pH3	EA	Silanediol, dimethyl-	4.537	13600505	685
EA_SPMEpH7	pH7	EA	Silanediol, dimethyl-	4.511	13670568	683
EC pH12_SPME	pH12	EC	Silanediol, dimethyl-	4.469	10275599	680
EC pH9_SPME	pH9	EC	Silanediol, dimethyl-	4.477	8938180	681
TSB pH12_SPME	pH12	TSB	2,2-Difluoroethanol, TBDMS derivative	4.456	7328482	680
TSB pH9_SPME	pH9	TSB	Silanediol, dimethyl-	4.461	21474063	680
5 mix pH7_SPME	pH7	5 mix	Silanediol, dimethyl-	4.558	16165887	687
5 mix pH9_SPME	pH9	5 mix	Silanediol, dimethyl-	4.617	14165811	691
EA pH9_SPME	pH9	EA	Silanediol, dimethyl-	4.55	18159870	686
CF pH5_SPME	pH5	CF	Ethanethioic acid, S-methyl ester	4.747	26053636	700

5 mix pH5_SPME	pH5	5 mix	Silanediol, dimethyl-	4.812	12891535	703
EA pH5_SPME	pH5	EA	Silanediol, dimethyl-	4.778	6928353	701
EC pH3_SPME	pH3	EC	Propanoic acid	4.828	8923811	703
EC pH5_SPME	pH5	EC	Propanoic acid	4.849	10328613	704
EC pH7_SPME	pH7	EC	Propanoic acid	4.815	3901967	703
EC pH9_SPME	pH9	EC	Propanoic acid	4.824	4385413	703
KO pH5_SPME	pH5	KO	Propanoic acid	4.837	13321423	704
PA pH3_SPME really	pH3	PA	2-Butanone, 3-hydroxy-	4.832	81337439	704
PA pH5_SPME	pH5	PA	2-Butanone, 3-hydroxy-	4.849	40496135	704
PA pH9_SPME	pH9	PA	2-Butanone, 3-hydroxy-	4.803	72913285	702
PA_SPMEpH7	pH7	PA	2-Butanone, 3-hydroxy-	4.837	72043941	704
5 mix pH12_SPME	pH12	5 mix	1-Butanamine, 3-methyl-	4.875	17640017	705
EA pH12_SPME	pH12	EA	1-Butanamine, 3-methyl-	4.862	24016407	705
EC pH12_SPME	pH12	EC	1-Butanamine, 3-methyl-	4.875	28960046	705
TSB pH12_SPME	pH12	TSB	1-Butanamine, 3-methyl-	4.862	23421691	705
PA pH3_SPME really	pH3	PA	Acetoin	5.221	6458212	720
5 mix pH3_SPME	pH3	5 mix	ISOAMYLALCOHOL	5.425	1.81E+08	729
5 mix pH12_SPME	pH12	5 mix	ISOAMYLALCOHOL	5.513	6.12E+08	732
5 mix pH5_SPME	pH5	5 mix	ISOAMYLALCOHOL	5.514	6.23E+08	733
CF pH12_SPME	pH12	CF	ISOAMYLALCOHOL	5.543	9.29E+08	734
CF pH3_SPME	pH3	CF	ISOAMYLALCOHOL	5.526	77604955	733
Cf_SPMEpH7	pH7	CF	ISOAMYLALCOHOL	5.463	3.65E+08	730
EA pH3_SPME	pH3	EA	ISOAMYLALCOHOL	5.484	21309822	731
EA_SPMEpH7	pH7	EA	Pyrazine	5.454	21512106	730
EC pH12_SPME	pH12	EC	ISOAMYLALCOHOL	5.513	7.96E+08	732
EC pH3_SPME	pH3	EC	ISOAMYLALCOHOL	5.496	2.03E+08	732
EC pH5_SPME	pH5	EC	ISOAMYLALCOHOL	5.517	8.14E+08	733
EC pH7_SPME	pH7	EC	1-Butanol, 2-methyl-	5.479	1.94E+08	731
EC pH9_SPME	pH9	EC	1-Butanol, 2-methyl-	5.522	1.73E+08	733
KO pH12_SPME	pH12	KO	ISOAMYLALCOHOL	5.509	7.45E+08	732
KO pH3_SPME	pH3	KO	ISOAMYLALCOHOL	5.492	3.97E+08	732
KO pH5_SPME	pH5	KO	ISOAMYLALCOHOL	5.513	7.83E+08	732
KO pH9_SPME	pH9	KO	ISOAMYLALCOHOL	5.488	2.75E+08	731
KO1_SPMEpH7	pH7	KO	ISOAMYLALCOHOL	5.501	7.71E+08	732
PA pH12_SPME	pH12	PA	ISOAMYLALCOHOL	5.53	8.87E+08	733

PA pH3_SPME really	pH3	PA	ISOAMYLALCOHOL	5.501	2.12E+08	732
PA pH5_SPME	pH5	PA	ISOAMYLALCOHOL	5.539	1.09E+09	734
PA pH9_SPME	pH9	PA	ISOAMYLALCOHOL	5.476	3.82E+08	731
PA_SPMEpH7	pH7	PA	ISOAMYLALCOHOL	5.488	5.9E+08	731
TSB pH12_SPME	pH12	TSB	ISOAMYLALCOHOL	5.539	2.63E+08	734
TSB pH3_SPME	pH3	TSB	Pyrazine	5.467	17389990	731
TSB_SPMEpH7	pH7	TSB	Pyrazine	5.488	18167393	731
5 mix pH7_SPME	pH7	5 mix	Disulfide, dimethyl	5.615	1.79E+08	737
5 mix pH9_SPME	pH9	5 mix	Disulfide, dimethyl	5.611	99933260	737
CF pH5_SPME	pH5	CF	ISOAMYLALCOHOL	5.559	1.02E+09	734
CF pH9_SPME	pH9	CF	ISOAMYLALCOHOL	5.573	1.72E+08	735
EA pH12_SPME	pH12	EA	ISOAMYLALCOHOL	5.577	2.16E+08	735
EA pH5_SPME	pH5	EA	Disulfide, dimethyl	5.611	1.57E+08	737
EA pH9_SPME	pH9	EA	Disulfide, dimethyl	5.636	2.91E+08	738
TSB pH5_SPME	pH5	TSB	Disulfide, dimethyl	5.606	1.8E+08	736
TSB pH9_SPME	pH9	TSB	1,3-Diazine	5.564	26054299	735
TSB pH3_SPME	pH3	TSB	Pentanoic acid	6.592	1847440	778
PA pH3_SPME really	pH3	PA	Propanoic acid, 2-methyl-	6.794	70257066	787
CF pH5_SPME	pH5	CF	Propanoic acid, 2-methyl-	7.001	24428138	796
EC pH5_SPME	pH5	EC	Propanoic acid, 2-methyl-	7.526	9827734	813
EC pH7_SPME	pH7	EC	Propanoic acid, 2-methyl-	7.526	14191137	813
CF pH9_SPME	pH9	CF	Cyclotrisiloxane, hexamethyl-	7.636	10594198	816
EA_SPMEpH7	pH7	EA	Cyclotrisiloxane, hexamethyl-	7.649	5305782	816
EC pH3_SPME	pH3	EC	Cyclotrisiloxane, hexamethyl-	7.56	2880279	814
EC pH9_SPME	pH9	EC	Cyclotrisiloxane, hexamethyl-	7.56	11849846	814
KO pH3_SPME	pH3	KO	Cyclotrisiloxane, hexamethyl-	7.611	4852782	815
5 mix pH12_SPME	pH12	5 mix	Pyrazine, methyl-	7.729		819
5 mix pH9_SPME	pH9	5 mix	Pyrazine, methyl-	7.729	19973385	819
CF pH9_SPME	pH9	CF	Pyrazine, methyl-	7.742	4006761	819
EA pH12_SPME	pH12	EA	Pyrazine, methyl-	7.725	55480629	819
EC pH12_SPME	pH12	EC	Pyrazine, methyl-	7.725	20941890	819
EC pH7_SPME	pH7	EC	Pyrazine, methyl-	7.725	6863514	819
EC pH9_SPME	pH9	EC	Pyrazine, methyl-	7.721	28702370	819
KO pH12_SPME	pH12	KO	Pyrazine, methyl-	7.716	44144821	818
KO pH9_SPME	pH9	KO	Cyclotrisiloxane, hexamethyl-	7.67	7081005	817

KO pH9_SPME	pH9	KO	Cyclotrisiloxane, hexamethyl-	7.729	5159163	819
PA pH12_SPME	pH12	PA	Cyclotrisiloxane, hexamethyl-	7.734	19832276	819
PA pH3_SPME really	pH3	PA	Cyclotrisiloxane, hexamethyl-	7.67	5302534	817
TSB pH12_SPME	pH12	TSB	Pyrazine, methyl-	7.729	48619595	819
5 mix pH3_SPME	pH3	5 mix	Pyrazine, methyl-	7.848	3813629	822
5 mix pH5_SPME	pH5	5 mix	Pyrazine, methyl-	7.793	35632970	821
CF pH12_SPME	pH12	CF	Pyrazine, methyl-	7.789	16227357	821
CF pH3_SPME	pH3	CF	Pyrazine, methyl-	7.814	4290189	821
CF pH5_SPME	pH5	CF	Pyrazine, methyl-	7.775	46705296	820
CF pH9_SPME	pH9	CF	Pyrazine, methyl-	7.805	8653190	821
Cf_SPMEpH7	pH7	CF	Pyrazine, methyl-	7.797	17912391	821
Cf_SPMEpH7	pH7	CF	Pyrazine, methyl-	7.818	9327869	822
EA pH3_SPME	pH3	EA	Pyrazine, methyl-	7.797	6802031	821
EA pH3_SPME	pH3	EA	Pyrazine, methyl-	7.822	6997089	822
EA pH5_SPME	pH5	EA	Pyrazine, methyl-	7.801	53494322	821
EA pH9_SPME	pH9	EA	Pyrazine, methyl-	7.818	12335428	822
EA_SPMEpH7	pH7	EA	Pyrazine, methyl-	7.788	15056563	821
EA_SPMEpH7	pH7	EA	Pyrazine, methyl-	7.814	20948836	821
EC pH12_SPME	pH12	EC	Pyrazine, methyl-	7.797	40102808	821
EC pH3_SPME	pH3	EC	Pyrazine, methyl-	7.775	5492471	820
EC pH3_SPME	pH3	EC	Pyrazine, methyl-	7.801	2156562	821
EC pH3_SPME	pH3	EC	Pyrazine, methyl-	7.818	2244832	822
EC pH5_SPME	pH5	EC	Pyrazine, methyl-	7.818	35675023	822
EC pH7_SPME	pH7	EC	Pyrazine, methyl-	7.78	5076299	820
EC pH7_SPME	pH7	EC	Pyrazine, methyl-	7.801	2912078	821
EC pH7_SPME	pH7	EC	Pyrazine, methyl-	7.818	5693433	822
KO pH3_SPME	pH3	KO	Pyrazine, methyl-	7.835	7161018	822
KO pH5_SPME	pH5	KO	Pyrazine, methyl-	7.805	35337728	821
KO pH9_SPME	pH9	KO	Pyrazine, methyl-	7.826	16734948	822
KO1_SPMEpH7	pH7	KO	Pyrazine, methyl-	7.797	17683077	821
PA pH12_SPME	pH12	PA	Pyrazine, methyl-	7.797	42693642	821
PA pH3_SPME really	pH3	PA	Pyrazine, methyl-	7.814	5265359	821
PA pH5_SPME	pH5	PA	Pyrazine, methyl-	7.805	44691436	821
PA_SPMEpH7	pH7	PA	Pyrazine, methyl-	7.81	14676418	821
TSB pH3_SPME	pH3	TSB	Pyrazine, methyl-	7.805	13566304	821
TSB pH5_SPME	pH5	TSB	Pyrazine, methyl-	7.792	42932810	821
TSB pH9_SPME	pH9	TSB	Pyrazine, methyl-	7.81	32235445	821
TSB_SPMEpH7	pH7	TSB	Pyrazine, methyl-	7.788	20852269	821

TSB_SPMEpH7	pH7	TSB	Pyrazine, methyl-	7.822	9866948	822[illegible]
5 mix pH7_SPME	pH7	5 mix	2-Furanmethanol	7.856		823[illegible]
CF pH9_SPME	pH9	CF	Pyrazine, methyl-	7.865	6829414	823[illegible]
Cf_SPMEpH7	pH7	CF	Pyrazine, methyl-	7.932	9488790	825[illegible]
EA pH3_SPME	pH3	EA	Pyrazine, methyl-	7.915	3358057	824[illegible]
EA pH9_SPME	pH9	EA	Pyrazine, methyl-	7.856	3278322	823[illegible]
EA pH9_SPME	pH9	EA	Pyrazine, methyl-	7.916	3573733	824[illegible]
EC pH3_SPME	pH3	EC	Pyrazine, methyl-	7.851	2686284	823[illegible]
EC pH3_SPME	pH3	EC	Pyrazine, methyl-	7.868	2232426	823[illegible]
EC pH3_SPME	pH3	EC	Pyrazine, methyl-	7.902	7901029	824[illegible]
EC pH7_SPME	pH7	EC	Pyrazine, methyl-	7.868	2679939	823[illegible]
EC pH7_SPME	pH7	EC	Pyrazine, methyl-	7.894	7405918	824[illegible]
EC pH9_SPME	pH9	EC	Pyrazine, methyl-	7.945	8932271	825[illegible]
KO pH3_SPME	pH3	KO	Pyrazine, methyl-	7.877	7202667	823[illegible]
KO pH9_SPME	pH9	KO	Pyrazine, methyl-	7.919	9877319	825[illegible]
PA pH3_SPME really	pH3	PA	Pyrazine, methyl-	7.928	3876418	825[illegible]
PA_SPMEpH7	pH7	PA	Pyrazine, methyl-	7.89	7935972	824[illegible]
TSB pH3_SPME	pH3	TSB	Pyrazine, methyl-	7.856	6618655	823[illegible]
TSB pH3_SPME	pH3	TSB	Pyrazine, methyl-	7.928	2514134	825[illegible]
TSB pH9_SPME	pH9	TSB	Pyrazine, methyl-	7.911	12899689	824[illegible]
TSB_SPMEpH7	pH7	TSB	Pyrazine, methyl-	7.902	14187557	824[illegible]
5 mix pH9_SPME	pH9	5 mix	Pyrazine, methyl-	7.974	6797551	826[illegible]
CF pH9_SPME	pH9	CF	Pyrazine, methyl-	7.953	2520106	826[illegible]
CF pH9_SPME	pH9	CF	Pyrazine, methyl-	8.008	3949777	827[illegible]
EA pH3_SPME	pH3	EA	Pyrazine, methyl-	7.958	4148646	826[illegible]
EA pH3_SPME	pH3	EA	Pyrazine, methyl-	8.017	3468225	827[illegible]
EA pH9_SPME	pH9	EA	Pyrazine, methyl-	7.979	5436432	826[illegible]
EA_SPMEpH7	pH7	EA	Pyrazine, methyl-	7.97	7578509	826[illegible]
EC pH3_SPME	pH3	EC	Pyrazine, methyl-	8.008	4999590	827[illegible]
EC pH7_SPME	pH7	EC	Pyrazine, methyl-	7.97	6704921	826[illegible]
KO pH3_SPME	pH3	KO	Pyrazine, methyl-	8.013	5290513	827[illegible]
PA pH3_SPME really	pH3	PA	Pyrazine, methyl-	8.029	4721540	828[illegible]
TSB pH3_SPME	pH3	TSB	Pyrazine, methyl-	7.979	5347647	826[illegible]
EA pH9_SPME	pH9	EA	Pyrazine, methyl-	8.622	5010025	846[illegible]
5 mix pH5_SPME	pH5	5 mix	Butanoic acid, 3-methyl-	8.736	11371592	849[illegible]
TSB pH3_SPME	pH3	TSB	Pentanoic acid	8.71	4398646	848[illegible]
TSB pH5_SPME	pH5	TSB	2-Furanmethanol	8.74	4924375	849[illegible]
TSB pH12_SPME	pH12	TSB	1H-Imidazole-2-methanol	8.778	5450208	850[illegible]

TSB<br>pH9_SPME	pH9	TSB	2-Furanmethanol	8.778	2905541	850
TSB_SPMEpH7	pH7	TSB	2-Furanmethanol	8.778	3393246	850
EC pH5_SPME	pH5	EC	Butanoic acid, 3-methyl-	8.892	21339881	854
TSB<br>pH3_SPME	pH3	TSB	Hexanoic acid, 2-methyl-	8.896	2172751	854
EA<br>pH12_SPME	pH12	EA	Octane, 4-methyl-	9.099	3872663	860
EC pH5_SPME	pH5	EC	Pentanoic acid	9.078	12017737	859
5 mix<br>pH5_SPME	pH5	5 mix	1-Butanol, 3-methyl-, acetate	9.577	21780130	874
CF pH5_SPME	pH5	CF	1-Butanol, 3-methyl-, acetate	9.568	23307905	874
EA pH5_SPME	pH5	EA	1-Butanol, 3-methyl-, acetate	9.573	10178843	874
EC pH5_SPME	pH5	EC	1-Butanol, 3-methyl-, acetate	9.581	15795979	875
EC pH7_SPME	pH7	EC	1-Butanol, 3-methyl-, acetate	9.581	12970682	875
EC pH9_SPME	pH9	EC	1-Butanol, 3-methyl-, acetate	9.581	3069802	875
KO pH3_SPME	pH3	KO	3-Methyl-hexanoic acid	9.598	31796573	875
KO pH5_SPME	pH5	KO	1-Butanol, 3-methyl-, acetate	9.577	28008058	874
KO pH9_SPME	pH9	KO	1-Butanol, 3-methyl-, acetate	9.577	17872250	874
KO1_SPMEpH7	pH7	KO	1-Butanol, 3-methyl-, acetate	9.573	1.61E+08	874
PA pH9_SPME	pH9	PA	1-Butanol, 3-methyl-, acetate	9.586	5061489	875
PA_SPMEpH7	pH7	PA	1-Butanol, 3-methyl-, acetate	9.565	38314424	874
TSB<br>pH5_SPME	pH5	TSB	1-Butanol, 3-methyl-, acetate	9.56	10031794	874
EC pH7_SPME	pH7	EC	2-Heptanone	10.004	5597640	887
EC pH9_SPME	pH9	EC	2-Heptanone	10	3780475	887
5 mix<br>pH12_SPME	pH12	5 mix	Oxime-, methoxy-phenyl-_	10.389	10203503	899
5 mix<br>pH9_SPME	pH9	5 mix	Oxime-, methoxy-phenyl-_	10.431	7652471	901
CF<br>pH12_SPME	pH12	CF	Oxime-, methoxy-phenyl-_	10.372	15538458	898
CF pH9_SPME	pH9	CF	Oxime-, methoxy-phenyl-_	10.393	15581946	899
Cf_SPMEpH7	pH7	CF	Oxime-, methoxy-phenyl-_	10.406	11546098	899
EA<br>pH12_SPME	pH12	EA	Oxime-, methoxy-phenyl-_	10.41	10789683	901
EA pH9_SPME	pH9	EA	Oxime-, methoxy-phenyl-_	10.394	7448145	899
EA_SPMEpH7	pH7	EA	Oxime-, methoxy-phenyl-_	10.406	5222242	899
EC<br>pH12_SPME	pH12	EC	Oxime-, methoxy-phenyl-_	10.41	10479773	901
EC pH7_SPME	pH7	EC	Oxime-, methoxy-phenyl-_	10.376	8284989	898
EC pH9_SPME	pH9	EC	Oxime-, methoxy-phenyl-_	10.41	9588830	901
KO<br>pH12_SPME	pH12	KO	Oxime-, methoxy-phenyl-_	10.41	10239472	901
KO pH9_SPME	pH9	KO	Oxime-, methoxy-phenyl-	10.397	8204045	899
KO1_SPMEpH7	pH7	KO	Oxime-, methoxy-phenyl-_	10.398	8084965	899
PA<br>pH12_SPME	pH12	PA	Oxime-, methoxy-phenyl-_	10.406	10273752	899
PA pH9_SPME	pH9	PA	Oxime-, methoxy-phenyl-_	10.398	10262813	899
PA_SPMEpH7	pH7	PA	Oxime-, methoxy-phenyl-_	10.394	7237612	899

TSB pH12_SPME	pH12	TSB	Oxime-, methoxy-phenyl-_	10.406	9339568	899
TSB pH3_SPME	pH3	TSB	Oxime-, methoxy-phenyl-_	10.372	1861502	898
TSB pH9_SPME	pH9	TSB	Oxime-, methoxy-phenyl-_	10.406	5311702	899
TSB_SPMEpH7	pH7	TSB	Oxime-, methoxy-phenyl-_	10.402	2657717	899
EA pH3_SPME	pH3	EA	Butanoic acid, 3-methyl-	10.596	3.26E+08	905
5 mix pH12_SPME	pH12	5 mix	Pyrazine, 2,5-dimethyl-	10.812	2.25E+08	911
5 mix pH5_SPME	pH5	5 mix	Pyrazine, 2,5-dimethyl-	10.821	2.08E+08	911
5 mix pH7_SPME	pH7	5 mix	Pyrazine, 2,5-dimethyl-	10.829	2.65E+08	912
5 mix pH9_SPME	pH9	5 mix	Pyrazine, 2,5-dimethyl-	10.824	2.56E+08	911
CF pH12_SPME	pH12	CF	Pyrazine, 2,6-dimethyl-	10.829	2.16E+08	912
CF pH5_SPME	pH5	CF	Pyrazine, 2,5-dimethyl-	10.816	2.09E+08	911
CF pH9_SPME	pH9	CF	Pyrazine, 2,5-dimethyl-	10.825	2.08E+08	911
Cf_SPMEpH7	pH7	CF	Pyrazine, 2,5-dimethyl-	10.808	2.33E+08	911
EA pH12_SPME	pH12	EA	Pyrazine, 2,6-dimethyl-	10.808	2.84E+08	911
EA pH5_SPME	pH5	EA	Pyrazine, 2,6-dimethyl-	10.812	2.43E+08	911
EA pH9_SPME	pH9	EA	Pyrazine, 2,5-dimethyl-	10.819	2.24E+08	911
EA_SPMEpH7	pH7	EA	Pyrazine, 2,6-dimethyl-	10.812	2.64E+08	911
EC pH12_SPME	pH12	EC	Pyrazine, 2,6-dimethyl-	10.816	2.73E+08	911
EC pH7_SPME	pH7	EC	Pyrazine, 2,5-dimethyl-	10.82	2.32E+08	911
EC pH9_SPME	pH9	EC	Pyrazine, 2,5-dimethyl-	10.825	2.57E+08	911
KO pH12_SPME	pH12	KO	Pyrazine, 2,6-dimethyl-	10.829	2.65E+08	912
KO pH5_SPME	pH5	KO	Pyrazine, 2,5-dimethyl-	10.82	2.14E+08	911
KO pH9_SPME	pH9	KO	Pyrazine, 2,5-dimethyl-	10.833	2.22E+08	912
KO1_SPMEpH7	pH7	KO	Pyrazine, 2,5-dimethyl-	10.829	2.25E+08	912
PA pH12_SPME	pH12	PA	Pyrazine, 2,6-dimethyl-	10.821	2.63E+08	911
PA pH3_SPME really	pH3	PA	Pentanoic acid	10.769	5351401	910
PA pH5_SPME	pH5	PA	Pyrazine, 2,5-dimethyl-	10.841	2.27E+08	912
PA pH9_SPME	pH9	PA	Pyrazine, 2,5-dimethyl-	10.825	2.39E+08	911
PA_SPMEpH7	pH7	PA	Pyrazine, 2,5-dimethyl-	10.816	2.37E+08	911
TSB pH12_SPME	pH12	TSB	Pyrazine, 2,6-dimethyl-	10.816	2.73E+08	911
TSB pH3_SPME	pH3	TSB	Pyrazine, 2,5-dimethyl-	10.837	1.81E+08	912
TSB pH5_SPME	pH5	TSB	Pyrazine, 2,5-dimethyl-	10.824	2.12E+08	911
TSB pH9_SPME	pH9	TSB	Pyrazine, 2,5-dimethyl-	10.825	2.54E+08	911

TSB_SPMEpH7	pH7	TSB	Pyrazine, 2,6-dimethyl-	10.829	2.57E+08	912
EA pH3_SPME	pH3	EA	Pyrazine, 2,5-dimethyl-	10.947	2.31E+08	915
EC pH5_SPME	pH5	EC	Pyrazine, 2,5-dimethyl-	10.875	2.27E+08	913
5 mix pH3_SPME	pH3	5 mix	Butanoic acid, 3-methyl-	10.973	37119384	915
EC pH3_SPME	pH3	EC	Butanoic acid, 3-methyl-	11.023	1.35E+08	917
KO pH3_SPME	pH3	KO	Butanoic acid, 3-methyl-	10.956	1.32E+08	915
KO pH3_SPME	pH3	KO	Butanoic acid, 3-methyl-	11.049	2.02E+08	917
PA pH3_SPME really	pH3	PA	Butanoic acid, 3-methyl-	11.044	3.66E+08	917
CF pH3_SPME	pH3	CF	Butanoic acid, 3-methyl-	11.082	1.62E+08	918
CF pH3_SPME	pH3	CF	Butanoic acid, 3-methyl-	11.103	13758374	919
CF pH3_SPME	pH3	CF	Butanoic acid, 3-methyl-	11.137	58569995	920
5 mix pH3_SPME	pH3	5 mix	Pyrazine, 2,5-dimethyl-	11.163	40566616	920
PA pH3_SPME really	pH3	PA	Pentanoic acid	11.433	1.88E+08	927
CF pH3_SPME	pH3	CF	Butanoic acid, 2-methyl-	11.45	7033006	928
EC pH3_SPME	pH3	EC	Butanoic acid, 2-methyl-	11.475	1.07E+08	928
PA pH3_SPME really	pH3	PA	Butanoic acid, 2-methyl-	11.679	1.44E+08	934
CF pH5_SPME	pH5	CF	Butanoic acid, 2-methyl-	11.919	19025707	940
EA pH5_SPME	pH5	EA	Butanoic acid, 2-methyl-	11.912	19656022	940
EA pH9_SPME	pH9	EA	Butanoic acid, 2-methyl-	11.899	6276214	939
TSB pH5_SPME	pH5	TSB	Thiopivalic acid	11.928	3166781	940
CF pH9_SPME	pH9	CF	Thiopivalic acid	12.203	2252894	947
TSB pH3_SPME	pH3	TSB	Hexanoic acid, 2-methylphenyl ester	12.279	2289324	949
TSB_SPMEpH7	pH7	TSB	3-Heptanone, 2,6-dimethyl-	12.288	3192633	950
5 mix pH12_SPME	pH12	5 mix	Benzaldehyde	12.711	29128013	961
5 mix pH3_SPME	pH3	5 mix	Benzaldehyde	12.736	28675280	961
5 mix pH9_SPME	pH9	5 mix	Benzaldehyde	12.672		960
CF pH12_SPME	pH12	CF	Benzaldehyde	12.719	16157979	961
CF pH9_SPME	pH9	CF	Benzaldehyde	12.719	7246002	961
EA pH12_SPME	pH12	EA	Benzaldehyde	12.719	27044635	961
EA pH5_SPME	pH5	EA	Benzaldehyde	12.745	7579288	962
EC pH12_SPME	pH12	EC	Benzaldehyde	12.698	29844447	960
EC pH9_SPME	pH9	EC	Benzaldehyde	12.744	2844206	962
KO pH12_SPME	pH12	KO	Benzaldehyde	12.723	27585115	961
KO pH9_SPME	pH9	KO	Benzaldehyde	12.736	8048072	961
PA pH12_SPME	pH12	PA	Benzaldehyde	12.715	26609327	961

PA pH9_SPME	pH9	PA	Benzaldehyde	12.736	19303711	961
TSB pH12_SPME	pH12	TSB	Benzaldehyde	12.715	23295628	961
TSB pH3_SPME	pH3	TSB	Benzaldehyde	12.728	40017642	961
TSB pH5_SPME	pH5	TSB	Benzaldehyde	12.719	36218263	961
TSB pH9_SPME	pH9	TSB	Benzaldehyde	12.728	18882139	961
TSB_SPMEpH7	pH7	TSB	Benzaldehyde	12.723	42273191	961
EA pH3_SPME	pH3	EA	Benzaldehyde	12.77	6301159	962
EA pH9_SPME	pH9	EA	Benzaldehyde	12.762	3072882	962
EA_SPMEpH7	pH7	EA	2-Heptanone, 5-methyl-	12.757	4608107	962
EC pH3_SPME	pH3	EC	2-Heptanone, 5-methyl-	12.757	2837086	962
5 mix pH5_SPME	pH5	5 mix	AMYLPROPIONATE	13.003	9826118	968
CF pH5_SPME	pH5	CF	AMYLPROPIONATE	12.993	26773408	968
EA pH5_SPME	pH5	EA	1-Butanol, 3-methyl-, propanoate	13.015	15326899	969
EA pH9_SPME	pH9	EA	1-Butanol, 3-methyl-, propanoate	13.041	3887323	969
EC pH5_SPME	pH5	EC	Propanoic acid, pentyl ester	13.03	94589611	969
EC pH7_SPME	pH7	EC	Propanoic acid, pentyl ester	13.023	17285680	969
EC pH9_SPME	pH9	EC	Propanoic acid, pentyl ester	13.019	3098813	969
KO pH5_SPME	pH5	KO	Propanoic acid, pentyl ester	13.036	1.58E+08	969
KO pH9_SPME	pH9	KO	AMYLPROPIONATE	13.027	8708896	969
PA pH5_SPME	pH5	PA	AMYLPROPIONATE	13.023	14140498	969
TSB pH3_SPME	pH3	TSB	Benzene, 1,2,4-trimethyl-	13.007	5051500	968
TSB pH5_SPME	pH5	TSB	1-Butanol, 3-methyl-, propanoate	13.011	25322739	969
EC pH3_SPME	pH3	EC	1-Butanol, 3-methyl-, propanoate	13.053	4558695	970
KO pH3_SPME	pH3	KO	Propanoic acid, pentyl ester	13.058	41671451	970
KO1_SPMEpH7	pH7	KO	Propanoic acid, pentyl ester	13.066	2.7E+08	970
5 mix pH12_SPME	pH12	5 mix	Phenol	13.315	11680824	976
5 mix pH5_SPME	pH5	5 mix	Phenol	13.408	2.82E+08	979
5 mix pH9_SPME	pH9	5 mix	Phenol	13.421	2.81E+08	979
CF pH3_SPME	pH3	CF	Phenol	13.446	2.91E+08	980
CF pH5_SPME	pH5	CF	Phenol	13.421	3.24E+08	979
CF pH9_SPME	pH9	CF	Phenol	13.362	1.62E+08	978
Cf_SPMEpH7	pH7	CF	Phenol	13.425	2.96E+08	979
TSB pH3_SPME	pH3	TSB	Hexanoic acid	13.413	2425574	979
5 mix pH12_SPME	pH12	5 mix	Cyclotetrasiloxane, octamethyl-	13.531	10788070	982
5 mix pH3_SPME	pH3	5 mix	Phenol	13.467	3.34E+08	980
5 mix pH7_SPME	pH7	5 mix	Phenol	13.455	3.58E+08	980

5 mix pH9_SPME	pH9	5 mix	siloxane	13.527	12491718	982
CF pH12_SPME	pH12	CF	Cyclotetrasiloxane, octamethyl-	13.531	12976895	982
CF pH9_SPME	pH9	CF	Cyclotetrasiloxane, octamethyl-	13.535	11975102	982
Cf_SPMEpH7	pH7	CF	Cyclotetrasiloxane, octamethyl-	13.518	13404343	982
EA pH12_SPME	pH12	EA	Cyclotetrasiloxane, octamethyl-	13.535	10237640	982
EA pH3_SPME	pH3	EA	Cyclotetrasiloxane, octamethyl-	13.535	4104444	982
EA pH5_SPME	pH5	EA	Cyclotetrasiloxane, octamethyl-	13.54	2567168	982
EA pH9_SPME	pH9	EA	Cyclotetrasiloxane, octamethyl-	13.536	5960261	982
EA_SPMEpH7	pH7	EA	Cyclotetrasiloxane, octamethyl-	13.539	4292024	982
EC pH12_SPME	pH12	EC	Cyclotetrasiloxane, octamethyl-	13.539	9975265	982
EC pH7_SPME	pH7	EC	Cyclotetrasiloxane, octamethyl-	13.535	5138845	982
EC pH9_SPME	pH9	EC	Cyclotetrasiloxane, octamethyl-	13.535	8837812	982
KO pH12_SPME	pH12	KO	Cyclotetrasiloxane, octamethyl-	13.531	10770008	982
KO pH9_SPME	pH9	KO	Cyclotetrasiloxane, octamethyl-	13.543	6646343	982
PA pH12_SPME	pH12	PA	Cyclotetrasiloxane, octamethyl-	13.535	9235704	982
PA pH3_SPME really	pH3	PA	Cyclotetrasiloxane, octamethyl-	13.548	6075962	983
PA pH9_SPME	pH9	PA	Cyclotetrasiloxane, octamethyl-	13.54	7130531	982
TSB pH12_SPME	pH12	TSB	Cyclotetrasiloxane, octamethyl-	13.54	8828210	982
TSB pH3_SPME	pH3	TSB	siloxane	13.531	4070774	982
TSB pH5_SPME	pH5	TSB	Cyclotetrasiloxane, octamethyl-	13.539	2665533	982
TSB pH9_SPME	pH9	TSB	Cyclotetrasiloxane, octamethyl-	13.54	5256887	982
TSB_SPMEpH7	pH7	TSB	Cyclotetrasiloxane, octamethyl-	13.535	3863252	982
EC pH3_SPME	pH3	EC	Cyclotetrasiloxane, octamethyl-	13.552	4720843	983
EC pH7_SPME	pH7	EC	Cyclotetrasiloxane, octamethyl-	13.624	17224929	985
EC pH9_SPME	pH9	EC	3-Octanone	13.611	8473543	984
KO pH3_SPME	pH3	KO	Cyclotetrasiloxane, octamethyl-	13.552	6394551	983
KO1_SPMEpH7	pH7	KO	Cyclotetrasiloxane, octamethyl-	13.633	7830988	985
5 mix pH5_SPME	pH5	5 mix	3-Octanone	13.696	20722319	986
EC pH3_SPME	pH3	EC	3-Octanone	13.662	12020156	986
KO pH3_SPME	pH3	KO	3-Octanone	13.692	8093204	986
5 mix pH12_SPME	pH12	5 mix	Benzene, 1,2,4-trimethyl-	13.954	15523056	993
PA pH9_SPME	pH9	PA	Mesitylene	13.967	11982968	994
TSB pH5_SPME	pH5	TSB	Benzene, 1,3,5-trimethyl-	13.954	2465030	993
5 mix pH12_SPME	pH12	5 mix	Pyrazine, 2-ethyl-5-methyl-	14.072	6708015	996

EA pH12_SPME	pH12	EA	Pyrazine, 2-ethyl-6-methyl-	14.06	4979046	996
EA pH5_SPME	pH5	EA	4-Pyridinamine, N,N-dimethyl-	14.073	2818220	996
TSB pH12_SPME	pH12	TSB	Pyrazine, 2-ethyl-5-methyl-	14.068	5366918	996
TSB pH5_SPME	pH5	TSB	1,3-Benzenediamine, 4-methyl-	14.076	2672613	996
5 mix pH12_SPME	pH12	5 mix	Pyrazine, 2-ethyl-3-methyl-	14.246	53622245	1001
CF pH12_SPME	pH12	CF	Pyrazine, 2-ethyl-3-methyl-	14.229	46438728	1000
EA pH12_SPME	pH12	EA	Pyrazine, 2-ethyl-3-methyl-	14.237	67426705	1001
EA pH5_SPME	pH5	EA	Pyrazine, 2-ethyl-3-methyl-	14.229	47593892	1000
EA pH9_SPME	pH9	EA	Pyrazine, 2-ethyl-3-methyl-	14.237	47063828	1001
EA_SPMEpH7	pH7	EA	Pyrazine, 2-ethyl-3-methyl-	14.237	54361541	1001
EC pH12_SPME	pH12	EC	Pyrazine, 2-ethyl-3-methyl-	14.237	70970510	1001
EC pH5_SPME	pH5	EC	Pyrazine, 2-ethyl-3-methyl-	14.241	46069475	1001
EC pH7_SPME	pH7	EC	Pyrazine, 2-ethyl-3-methyl-	14.233	42903009	1000
EC pH9_SPME	pH9	EC	Pyrazine, 2-ethyl-3-methyl-	14.237	50569123	1001
KO pH12_SPME	pH12	KO	Pyrazine, 2-ethyl-3-methyl-	14.245	66415599	1001
KO pH5_SPME	pH5	KO	Pyrazine, 2-ethyl-3-methyl-	14.237	45122618	1001
PA pH12_SPME	pH12	PA	Pyrazine, 2-ethyl-3-methyl-	14.233	70441449	1000
PA pH5_SPME	pH5	PA	Pyrazine, 2-ethyl-3-methyl-	14.237	49579046	1001
PA pH9_SPME	pH9	PA	Pyrazine, 2-ethyl-3-methyl-	14.242	60224340	1001
PA_SPMEpH7	pH7	PA	Pyrazine, 2-ethyl-3-methyl-	14.235	53733413	1001
TSB pH12_SPME	pH12	TSB	Pyrazine, 2-ethyl-3-methyl-	14.233	68045260	1000
TSB pH5_SPME	pH5	TSB	Pyrazine, 2-ethyl-3-methyl-	14.241	41895436	1001
TSB pH9_SPME	pH9	TSB	Pyrazine, 2-ethyl-3-methyl-	14.237	48849629	1001
TSB_SPMEpH7	pH7	TSB	Pyrazine, 2-ethyl-3-methyl-	14.233	49036093	1000
5 mix pH5_SPME	pH5	5 mix	Pyrazine, 2-ethyl-3-methyl-	14.271	40081044	1001
5 mix pH7_SPME	pH7	5 mix	Pyrazine, 2-ethyl-3-methyl-	14.292	21014398	1002
5 mix pH7_SPME	pH7	5 mix	Pyrazine, 2-ethyl-5-methyl-	14.33	29545899	1003
5 mix pH9_SPME	pH9	5 mix	Pyrazine, 2-ethyl-3-methyl-	14.317	51621527	1003
CF pH5_SPME	pH5	CF	Pyrazine, 2-ethyl-3-methyl-	14.27	25574391	1001
CF pH5_SPME	pH5	CF	Pyrazine, 2-ethyl-5-methyl-	14.309	17379856	1002
CF pH9_SPME	pH9	CF	Pyrazine, 2-ethyl-3-methyl-	14.296	35526520	1002
Cf_SPMEpH7	pH7	CF	Pyrazine, 2-ethyl-3-methyl-	14.279	17273285	1002
Cf_SPMEpH7	pH7	CF	Pyrazine, 2-ethyl-5-methyl-	14.313	23873077	1003
EA pH3_SPME	pH3	EA	Pyrazine, 2-ethyl-3-methyl-	14.301	39543834	1002

KO pH9_SPME	pH9	KO	Pyrazine, 2-ethyl-3-methyl-	14.25	45107489	1001
KO1_SPMEpH7	pH7	KO	Pyrazine, 2-ethyl-3-methyl-	14.25	48445081	1001
TSB pH3_SPME	pH3	TSB	Pyrazine, 2-ethyl-3-methyl-	14.263	25652193	1001
5 mix pH3_SPME	pH3	5 mix	Pyrazine, 2-ethyl-3-methyl-	14.427	31672126	1005
CF pH3_SPME	pH3	CF	Pyrazine, 2-ethyl-3-methyl-	14.444	29823717	1006
EC pH3_SPME	pH3	EC	Pyrazine, 2-ethyl-3-methyl-	14.448	43973626	1006
KO pH3_SPME	pH3	KO	Pyrazine, 2-ethyl-3-methyl-	14.406	41697102	1005
PA pH3_SPME really	pH3	PA	Pyrazine, 2-ethyl-3-methyl-	14.478	24932867	1007
CF pH5_SPME	pH5	CF	Undecane	14.698	12517854	1012
EA pH12_SPME	pH12	EA	Nonane, 2,5-dimethyl-	14.707	4100397	1013
TSB pH12_SPME	pH12	TSB	Nonane, 2,5-dimethyl-	14.707	4306291	1013
TSB pH5_SPME	pH5	TSB	Nonane, 2,5-dimethyl-	14.706	2497278	1013
EA pH12_SPME	pH12	EA	Nonane, 2,5-dimethyl-	14.914	5187657	1018
TSB pH12_SPME	pH12	TSB	Decane, 5-methyl-	14.927	4987424	1018
TSB pH5_SPME	pH5	TSB	4-Hydroxy-4-methylhex-5-enoic acid, tert.-butyl ester	14.918	2341666	1018
5 mix pH12_SPME	pH12	5 mix	Benzene, 1,3,5-trimethyl-	14.998	6967055	1020
PA pH9_SPME	pH9	PA	Benzene, 1,3,5-trimethyl-	14.999	4297156	1020
CF pH5_SPME	pH5	CF	Decane, 4-methyl-	15.061	13467691	1022
EA pH12_SPME	pH12	EA	Decane, 4-methyl-	15.062	8054141	1022
EA pH5_SPME	pH5	EA	Decane, 4-methyl-	15.058	5908187	1022
EC pH12_SPME	pH12	EC	Decane, 4-methyl-	15.062	9551784	1022
TSB pH12_SPME	pH12	TSB	Decane, 4-methyl-	15.066	7563539	1022
TSB pH5_SPME	pH5	TSB	Decane, 4-methyl-	15.062	5181510	1022
TSB pH5_SPME	pH5	TSB	Cyclohexene, 1-methyl-4-(1-methylethenyl)-, (R)-	15.358	2888525	1029
5 mix pH3_SPME	pH3	5 mix	Benzeneacetaldehyde	15.92	8721826	1044
CF pH3_SPME	pH3	CF	Benzeneacetaldehyde	15.916	3906198	1044
EA pH12_SPME	pH12	EA	1-Butanamine, 3-methyl-N-(3-methylbutylidene)-	15.891	4785244	1043
EC pH3_SPME	pH3	EC	Benzeneacetaldehyde	15.911	3337825	1044
KO pH3_SPME	pH3	KO	Benzeneacetaldehyde	15.918	22449194	1044
TSB pH12_SPME	pH12	TSB	3-methylbutyl-(3-methylbutylidene)amine	15.886	7974992	1043
TSB pH3_SPME	pH3	TSB	Benzeneacetaldehyde	15.899	5001139	1043

TSB pH5_SPME	pH5	TSB	Benzeneacetaldehyde	15.895	5328576	1043
5 mix pH5_SPME	pH5	5 mix	Nonane, 4,5-dimethyl-	16.329	8423323	1055
CF pH12_SPME	pH12	CF	Nonane, 4,5-dimethyl-	16.322	19927660	1054
CF pH5_SPME	pH5	CF	Nonane, 4,5-dimethyl-	16.328	37563197	1055
EA pH12_SPME	pH12	EA	Nonane, 4,5-dimethyl-	16.331	28789090	1055
EA pH5_SPME	pH5	EA	Nonane, 4,5-dimethyl-	16.326	15095206	1054
EA pH9_SPME	pH9	EA	Nonane, 4,5-dimethyl-	16.288	2969168	1053
EC pH12_SPME	pH12	EC	Nonane, 4,5-dimethyl-	16.33	37227130	1055
KO pH12_SPME	pH12	KO	Nonane, 4,5-dimethyl-	16.33	27578376	1055
KO pH5_SPME	pH5	KO	Nonane, 4,5-dimethyl-	16.33	14206383	1055
PA pH12_SPME	pH12	PA	Nonane, 4,5-dimethyl-	16.331	28772698	1055
PA pH9_SPME	pH9	PA	Nonane, 4,5-dimethyl-	16.305	9719697	1054
TSB pH12_SPME	pH12	TSB	Nonane, 4,5-dimethyl-	16.33	26091029	1055
TSB pH5_SPME	pH5	TSB	Nonane, 4,5-dimethyl-	16.326	12769666	1054
CF pH5_SPME	pH5	CF	Nonane, 4,5-dimethyl-	16.537	10363714	1060
EA pH12_SPME	pH12	EA	Decane, 4-methyl-	16.545	8550219	1060
EA pH5_SPME	pH5	EA	Nonane, 4,5-dimethyl-	16.542	3891418	1060
EC pH12_SPME	pH12	EC	Undecane, 5-methyl-	16.546	11504512	1060
KO pH12_SPME	pH12	KO	Undecane, 5-methyl-	16.546	8152590	1060
TSB pH12_SPME	pH12	TSB	Nonane, 4,5-dimethyl-	16.542	7826006	1060
TSB pH5_SPME	pH5	TSB	Dodecane, 4,6-dimethyl-	16.546	3553657	1060
EA_SPMEpH7	pH7	EA	Dodecane, 4,6-dimethyl-	16.728	4855756	1065
TSB pH12_SPME	pH12	TSB	Acetophenone	16.732	3211850	1065
TSB pH3_SPME	pH3	TSB	1-phenyl-ethanone	16.736	4162753	1065
TSB pH9_SPME	pH9	TSB	Acetophenone	16.732	5008401	1065
TSB_SPMEpH7	pH7	TSB	1-phenyl-ethanone	16.728	5079302	1065
CF pH9_SPME	pH9	CF	Cyclotrisiloxane, hexamethyl-	16.808	6750558	1067
Cf_SPMEpH7	pH7	CF	Cyclotrisiloxane, hexamethyl-	16.791	4110755	1066
EA pH9_SPME	pH9	EA	Cyclotrisiloxane, hexamethyl-	16.787	4756333	1066
EC pH7_SPME	pH7	EC	Cyclotrisiloxane, hexamethyl-	16.791	2883570	1066
5 mix pH3_SPME	pH3	5 mix	p-cresol	16.982	3952361	1071
EC pH3_SPME	pH3	EC	p-Cresol	16.994	4055111	1072

EC pH7_SPME	pH7	EC	p-Cresol	16.973	2613152	1071
KO pH3_SPME	pH3	KO	p-Cresol	16.994	9428496	1072
KO pH9_SPME	pH9	KO	p-Cresol	16.984	5171681	1071
KO1_SPMEpH7	pH7	KO	p-Cresol	16.986	9815770	1071
5 mix pH12_SPME	pH12	5 mix	Pyrazine, 3-ethyl-2,5-dimethyl-	17.117	24652173	1075
5 mix pH3_SPME	pH3	5 mix	Pyrazine, 3-ethyl-2,5-dimethyl-	17.146	9649054	1076
5 mix pH5_SPME	pH5	5 mix	Pyrazine, 3-ethyl-2,5-dimethyl-	17.13	18026875	1075
5 mix pH7_SPME	pH7	5 mix	Pyrazine, 3-ethyl-2,5-dimethyl-	17.142	21590422	1075
5 mix pH9_SPME	pH9	5 mix	Pyrazine, 3-ethyl-2,5-dimethyl-	17.129	20281209	1075
CF pH12_SPME	pH12	CF	Pyrazine, 3-ethyl-2,5-dimethyl-	17.107	21670815	1075
CF pH3_SPME	pH3	CF	Pyrazine, 3-ethyl-2,5-dimethyl-	17.146	9577272	1076
CF pH5_SPME	pH5	CF	Pyrazine, 3-ethyl-2,5-dimethyl-	17.121	21486563	1075
CF pH9_SPME	pH9	CF	Pyrazine, 3-ethyl-2,5-dimethyl-	17.121	11534435	1075
Cf_SPMEpH7	pH7	CF	Pyrazine, 3-ethyl-2,5-dimethyl-	17.121	16141460	1075
EA pH12_SPME	pH12	EA	Pyrazine, 3-ethyl-2,5-dimethyl-	17.111	26657460	1075
EA pH3_SPME	pH3	EA	Pyrazine, 3-ethyl-2,5-dimethyl-	17.121	14333907	1075
EA pH5_SPME	pH5	EA	Pyrazine, 3-ethyl-2,5-dimethyl-	17.109	17460007	1075
EA pH9_SPME	pH9	EA	Pyrazine, 3-ethyl-2,5-dimethyl-	17.109	17968140	1075
EA_SPMEpH7	pH7	EA	Pyrazine, 3-ethyl-2,5-dimethyl-	17.109	22543877	1075
EC pH12_SPME	pH12	EC	Pyrazine, 3-ethyl-2,5-dimethyl-	17.108	29197969	1075
EC pH5_SPME	pH5	EC	Pyrazine, 3-ethyl-2,5-dimethyl-	17.114	18471593	1075
EC pH7_SPME	pH7	EC	Pyrazine, 3-ethyl-2,5-dimethyl-	17.104	13326290	1075
EC pH9_SPME	pH9	EC	Pyrazine, 3-ethyl-2,5-dimethyl-	17.108	15614138	1075
KO pH12_SPME	pH12	KO	Pyrazine, 3-ethyl-2,5-dimethyl-	17.116	27845032	1075
KO pH5_SPME	pH5	KO	Pyrazine, 3-ethyl-2,5-dimethyl-	17.117	18011886	1075
KO pH9_SPME	pH9	KO	Pyrazine, 3-ethyl-2,5-dimethyl-	17.117	19377087	1075
KO1_SPMEpH7	pH7	KO	Pyrazine, 3-ethyl-2,5-dimethyl-	17.117	23373232	1075
PA pH12_SPME	pH12	PA	Pyrazine, 3-ethyl-2,5-dimethyl-	17.113	27724702	1075
PA pH5_SPME	pH5	PA	Pyrazine, 3-ethyl-2,5-dimethyl-	17.108	19487813	1075
PA pH9_SPME	pH9	PA	Pyrazine, 3-ethyl-2,5-dimethyl-	17.117	29664357	1075
PA_SPMEpH7	pH7	PA	Pyrazine, 3-ethyl-2,5-dimethyl-	17.109	30726036	1075
TSB pH12_SPME	pH12	TSB	Pyrazine, 3-ethyl-2,5-dimethyl-	17.111	28548401	1075
TSB pH3_SPME	pH3	TSB	Pyrazine, 3-ethyl-2,5-dimethyl-	17.117	7179707	1075
TSB pH5_SPME	pH5	TSB	Pyrazine, 3-ethyl-2,5-dimethyl-	17.113	16251621	1075
TSB pH9_SPME	pH9	TSB	Pyrazine, 3-ethyl-2,5-dimethyl-	17.113	20832499	1075
TSB_SPMEpH7	pH7	TSB	Pyrazine, 3-ethyl-2,5-dimethyl-	17.107	22875318	1075

EC pH3_SPME	pH3	EC	Pyrazine, 3-ethyl-2,5-dimethyl-	17.159	12970947	1076
KO pH3_SPME	pH3	KO	Pyrazine, 3-ethyl-2,5-dimethyl-	17.16	17074274	1076
PA pH3_SPME really	pH3	PA	Pyrazine, 3-ethyl-2,5-dimethyl-	17.16	9764775	1076
TSB pH5_SPME	pH5	TSB	3-Methyl-2-thiophenecarboxaldehyde	17.438	4044420	1083
PA pH9_SPME	pH9	PA	Pyrazine, 2-ethyl-3,5-dimethyl-	17.455	5273354	1084
TSB pH3_SPME	pH3	TSB	3-Methyl-2-thiophenecarboxaldehyde	17.451	2249863	1083
TSB_SPMEpH7	pH7	TSB	3-Methyl-2-thiophenecarboxaldehyde	17.451	3005983	1083
5 mix pH12_SPME	pH12	5 mix	2-Nonanone	17.698		1090
5 mix pH5_SPME	pH5	5 mix	2-Nonanone	17.709	5803749	1090
CF pH5_SPME	pH5	CF	2-Nonanone	17.695	11523266	1090
Cf_SPMEpH7	pH7	CF	2-Nonanone	17.696	3982309	1090
EC pH12_SPME	pH12	EC	2-Nonanone	17.696	14738619	1090
EC pH3_SPME	pH3	EC	2-Nonanone	17.713	9345385	1090
EC pH7_SPME	pH7	EC	2-Nonanone	17.692	14230947	1090
EC pH9_SPME	pH9	EC	2-Nonanone	17.692	8224129	1090
KO pH3_SPME	pH3	KO	2-Nonanone	17.713	6171379	1090
KO1_SPMEpH7	pH7	KO	2-Nonanone	17.701	9498167	1090
PA pH3_SPME really	pH3	PA	2-Nonanone	17.717	13437096	1090
PA pH9_SPME	pH9	PA	2-Nonanone	17.701	14510080	1090
PA_SPMEpH7	pH7	PA	2-Nonanone	17.701	43475427	1090
CF pH9_SPME	pH9	CF	2-Nonanone	17.933	5092604	1096
Cf_SPMEpH7	pH7	CF	2-Nonanone	17.931	5620009	1096
EA pH12_SPME	pH12	EA	2-Nonanone	17.933	5424171	1096
EA pH3_SPME	pH3	EA	2-Nonanone	17.937	3394690	1096
EA pH9_SPME	pH9	EA	2-Nonanone	17.933	4683494	1096
EA_SPMEpH7	pH7	EA	2-Nonanone	17.931	5809037	1096
EC pH7_SPME	pH7	EC	2-Nonanone	17.93	3488828	1096
EC pH9_SPME	pH9	EC	2-Nonanone	17.929	4840830	1096
KO pH9_SPME	pH9	KO	2-Nonanone	17.945	4990114	1096
PA pH9_SPME	pH9	PA	2-Nonanone	17.938	5308907	1096
TSB pH12_SPME	pH12	TSB	2-Hydroxy-alpha-(p-methoxybenzylidene)-5-methylacetophenone	17.934	5056515	1096
TSB pH3_SPME	pH3	TSB	Pyrazine, 2-methyl-5-(2-propenyl)-	17.942	2668468	1096
TSB pH9_SPME	pH9	TSB	Pyrazine, 2-methyl-5-(2-propenyl)-	17.937	4658502	1096
TSB_SPMEpH7	pH7	TSB	Pyrazine, 2-methyl-5-(2-propenyl)-	17.929	5476500	1096
EC pH3_SPME	pH3	EC	Pyrazine, 2-methyl-5-(2-propenyl)-	17.958	3066873	1097
CF pH5_SPME	pH5	CF	Nonane, 4,5-dimethyl-	18.059	10565912	1099
EA pH12_SPME	pH12	EA	Nonane, 4,5-dimethyl-	18.064	11348828	1099
EA pH5_SPME	pH5	EA	Nonane, 4,5-dimethyl-	18.064	3200430	1099

EC pH12_SPME	pH12	EC	Nonane, 4,5-dimethyl-	18.066	13170356	1099
KO pH12_SPME	pH12	KO	Nonane, 4,5-dimethyl-	18.064	10990417	1099
PA pH12_SPME	pH12	PA	Nonane, 4,5-dimethyl-	18.069	11354905	1099
TSB pH12_SPME	pH12	TSB	Nonane, 4,5-dimethyl-	18.064	8590795	1099
TSB pH5_SPME	pH5	TSB	Nonane, 4,5-dimethyl-	18.064	2975521	1099
EA pH12_SPME	pH12	EA	Nonane, 4,5-dimethyl-	18.277	3193932	1105
TSB pH3_SPME	pH3	TSB	Nonanal	18.252	5201274	1104
TSB pH5_SPME	pH5	TSB	Nonanal	18.25	3288455	1104
5 mix pH9_SPME	pH9	5 mix	2-Phenylethanol	18.538	54954793	1112
CF pH12_SPME	pH12	CF	2-Phenylethanol	18.516	14185898	1111
CF pH3_SPME	pH3	CF	2-Phenylethanol	18.529	18581078	1112
CF pH5_SPME	pH5	CF	2-Phenylethanol	18.52	32298305	1111
CF pH9_SPME	pH9	CF	2-Phenylethanol	18.525	17068564	1111
Cf_SPMEpH7	pH7	CF	2-Phenylethanol	18.517	21506366	1111
EC pH5_SPME	pH5	EC	2-Phenylethanol	18.546	85251091	1112
EC pH7_SPME	pH7	EC	2-Phenylethanol	18.525	45394311	1111
EC pH9_SPME	pH9	EC	2-Phenylethanol	18.538	61673791	1112
PA pH9_SPME	pH9	PA	2-Phenylethanol	18.538	55417136	1112
5 mix pH12_SPME	pH12	5 mix	2-Phenylethanol	18.584	1.55E+08	1113
5 mix pH3_SPME	pH3	5 mix	2-Phenylethanol	18.572	92603869	1113
5 mix pH5_SPME	pH5	5 mix	2-Phenylethanol	18.593	1.83E+08	1113
5 mix pH7_SPME	pH7	5 mix	2-Phenylethanol	18.55	57815547	1112
EC pH12_SPME	pH12	EC	2-Phenylethanol	18.554	95914349	1112
EC pH3_SPME	pH3	EC	2-Phenylethanol	18.567	69241246	1113
KO pH12_SPME	pH12	KO	2-Phenylethanol	18.58	1.53E+08	1113
KO pH3_SPME	pH3	KO	2-Phenylethanol	18.584	1.05E+08	1113
KO pH5_SPME	pH5	KO	2-Phenylethanol	18.58	1.58E+08	1113
KO pH9_SPME	pH9	KO	2-Phenylethanol	18.55	70487199	1112
KO1_SPMEpH7	pH7	KO	2-Phenylethanol	18.58	1.38E+08	1113
PA pH12_SPME	pH12	PA	2-Phenylethanol	18.584	1.56E+08	1113
PA pH3_SPME really	pH3	PA	2-Phenylethanol	18.567	59577933	1113
PA pH5_SPME	pH5	PA	2-Phenylethanol	18.584	1.74E+08	1113

PA_SPMEpH7	pH7	PA	2-Phenylethanol	18.563	1.13E+08	1113
5 mix pH12_SPME	pH12	5 mix	1,3,8-p-Menthatriene	18.808		1119
5 mix pH12_SPME	pH12	5 mix	Benzyl methyl ketone	19.071		1126
EA pH12_SPME	pH12	EA	Benzyl methyl ketone	19.054	3455887	1126
EA pH3_SPME	pH3	EA	Benzyl methyl ketone	19.058	23274690	1126
EA pH5_SPME	pH5	EA	Benzyl methyl ketone	19.054	7757483	1126
EA pH9_SPME	pH9	EA	Benzyl methyl ketone	19.062	25515244	1126
EA_SPMEpH7	pH7	EA	Benzyl methyl ketone	19.058	23342017	1126
CF pH9_SPME	pH9	CF	Cyclopentasiloxane, decamethyl-	19.388	4195034	1134
EA pH12_SPME	pH12	EA	Cyclopentasiloxane, decamethyl-	19.383	4809406	1134
EC pH7_SPME	pH7	EC	Cyclopentasiloxane, decamethyl-	19.383	2764174	1134
EC pH9_SPME	pH9	EC	Cyclopentasiloxane, decamethyl-	19.383	3581701	1134
TSB pH12_SPME	pH12	TSB	Cyclopentasiloxane, decamethyl-	19.384	4170620	1134
EA pH5_SPME	pH5	EA	Cyclopentasiloxane, decamethyl-	20.149	2739635	1155
CF pH3_SPME	pH3	CF	Cyclohexanone, 5-methyl-2-(1-methylethyl)-, trans-	20.225	8729640	1157
EA pH3_SPME	pH3	EA	Cyclohexanone, 5-methyl-2-(1-methylethyl)-, trans-	20.225	6360605	1157
KO pH3_SPME	pH3	KO	Cyclohexanone, 5-methyl-2-(1-methylethyl)-, trans-	20.236	7100412	1157
PA pH3_SPME really	pH3	PA	Cyclohexanone, 5-methyl-2-(1-methylethyl)-, trans-	20.232	3983365	1157
TSB pH3_SPME	pH3	TSB	Cyclohexanone, 5-methyl-2-(1-methylethyl)-, trans-	20.229	8834121	1157
EA pH12_SPME	pH12	EA	6-Dodecanone	20.339	4916663	1160
EA pH3_SPME	pH3	EA	2-Decanone	20.343	5930521	1160
EA pH5_SPME	pH5	EA	2-Decanone	20.344	7211189	1160
EA pH9_SPME	pH9	EA	2-Decanone	20.344	3542646	1160
EA_SPMEpH7	pH7	EA	2-Decanone	20.343	6879498	1160
EA pH12_SPME	pH12	EA	Undecane, 2-methyl-	20.496	3526728	1164
5 mix pH12_SPME	pH12	5 mix	2-Isoamylpyrazine	20.986		1177
CF pH3_SPME	pH3	CF	Cyclohexanol, 5-methyl-2-(1-methylethyl)-	21.041	32268637	1178
EA pH3_SPME	pH3	EA	Cyclohexanol, 5-methyl-2-(1-methylethyl)-	21.033	17585701	1178
EC pH3_SPME	pH3	EC	Cyclohexanol, 5-methyl-2-(1-methylethyl)-, (1.alpha.,2.alpha.,5.beta.)-	21.041	5533497	1178
PA pH3_SPME really	pH3	PA	Cyclohexanol, 5-methyl-2-(1-methylethyl)-	21.041	10452392	1178
TSB pH3_SPME	pH3	TSB	Cyclohexanol, 5-methyl-2-(1-methylethyl)-	21.041	27325860	1178
KO pH3_SPME	pH3	KO	Levomenthol	21.05	29789338	1179
5 mix pH12_SPME	pH12	5 mix	meta-Methoxybenzenethiol	21.358		1187

5 mix pH12_SPME	pH12	5 mix	Pyrazine, 2-methyl-6-(2-propenyl)-	21.709		1196
EA pH12_SPME	pH12	EA	Dodecane	21.84	4064488	1199
CF pH9_SPME	pH9	CF	Cyclotetrasiloxane, octamethyl-	22.31	4309872	1213
EA pH12_SPME	pH12	EA	Cyclotetrasiloxane, octamethyl-	22.287	5901833	1212
TSB pH12_SPME	pH12	TSB	Cyclotetrasiloxane, octamethyl-	22.284	3919755	1211
5 mix pH12_SPME	pH12	5 mix	Furan, 3-phenyl-	22.644	8112590	1222
5 mix pH5_SPME	pH5	5 mix	Furan, 3-phenyl-	22.643	16482647	1222
CF pH5_SPME	pH5	CF	Furan, 3-phenyl-	22.639	14519574	1222
EA pH12_SPME	pH12	EA	2-naphthalenol	22.644	8039672	1222
EA pH5_SPME	pH5	EA	Furan, 3-phenyl-	22.644	7195661	1222
EC pH12_SPME	pH12	EC	Furan, 3-phenyl-	22.639	13328873	1222
EC pH3_SPME	pH3	EC	Furan, 3-phenyl-	22.648	3401149	1222
EC pH7_SPME	pH7	EC	Furan, 3-phenyl-	22.644	5328862	1222
EC pH9_SPME	pH9	EC	Furan, 3-phenyl-	22.64	3418196	1222
KO pH5_SPME	pH5	KO	Furan, 3-phenyl-	22.644	12086206	1222
KO1_SPMEpH7	pH7	KO	Furan, 3-phenyl-	22.644	15939307	1222
PA_SPMEpH7	pH7	PA	Furan, 3-phenyl-	22.644	20097408	1222
TSB pH12_SPME	pH12	TSB	Furan, 3-phenyl-	22.644	7906216	1222
TSB pH5_SPME	pH5	TSB	Furan, 3-phenyl-	22.648	11484180	1222
TSB_SPMEpH7	pH7	TSB	Furan, 3-phenyl-	22.644	11765244	1222
EA_SPMEpH7	pH7	EA	Furan, 3-phenyl-	22.652	2730400	1222
KO pH3_SPME	pH3	KO	Furan, 3-phenyl-	22.652	8417433	1222
KO pH9_SPME	pH9	KO	Furan, 3-phenyl-	22.652	6340155	1222
PA pH3_SPME really	pH3	PA	Furan, 3-phenyl-	22.651	5630352	1222
PA pH9_SPME	pH9	PA	Furan, 3-phenyl-	22.651	6240229	1222
TSB pH3_SPME	pH3	TSB	Furan, 3-phenyl-	22.655	4335232	1222
5 mix pH12_SPME	pH12	5 mix	2-Isoamyl-6-methylpyrazine	23.57		1248
EA pH12_SPME	pH12	EA	Dodecane, 4,6-dimethyl-	23.705	3753859	1252
TSB pH12_SPME	pH12	TSB	Dodecane, 4,6-dimethyl-	23.705	3111019	1252
EA pH12_SPME	pH12	EA	Dodecane, 4,6-dimethyl-	24.037	4457050	1261
TSB pH12_SPME	pH12	TSB	Dodecane, 4,6-dimethyl-	24.035	3880700	1261
EA pH12_SPME	pH12	EA	Dodecane, 4,6-dimethyl-	24.475	8708898	1274

EA pH5_SPME	pH5	EA	Dodecane, 4,6-dimethyl-	24.475	3971782	1274
EC pH12_SPME	pH12	EC	Pentadecane	24.479	18877381	1274
KO pH12_SPME	pH12	KO	Dodecane, 4,6-dimethyl-	24.483	17046706	1274
PA pH12_SPME	pH12	PA	Dodecane, 4,6-dimethyl-	24.479	17106867	1274
TSB pH12_SPME	pH12	TSB	Pentadecane	24.475	12645037	1274
TSB pH5_SPME	pH5	TSB	Pentadecane	24.479	4167201	1274
CF pH3_SPME	pH3	CF	Cyclohexanol, 5-methyl-2-(1-methylethyl)-, acetate	25.02	6552325	1289
TSB pH3_SPME	pH3	TSB	Cyclohexanol, 5-methyl-2-(1-methylethyl)-, acetate, (1.alpha.,2.beta.,5.alpha.)-	25.029	6094210	1290
CF pH5_SPME	pH5	CF	2-Undecanone	25.104	67278954	1292
Cf_SPMEpH7	pH7	CF	2-Undecanone	25.092	7094287	1291
EA pH12_SPME	pH12	EA	indole	25.092	8770792	1291
EA pH3_SPME	pH3	EA	indole	25.101	10999862	1292
EA pH5_SPME	pH5	EA	indole	25.093	12119989	1291
EA pH9_SPME	pH9	EA	indole	25.105	9314119	1292
EA_SPMEpH7	pH7	EA	indole	25.113	3718399	1292
EC pH12_SPME	pH12	EC	indole	25.092	16405792	1291
EC pH3_SPME	pH3	EC	indole	25.096	37074225	1291
EC pH5_SPME	pH5	EC	2-Undecanone	25.092	15884894	1291
EC pH7_SPME	pH7	EC	indole	25.088	65406750	1291
EC pH9_SPME	pH9	EC	indole	25.088	76489143	1291
PA pH3_SPME really	pH3	PA	2-Undecanone	25.109	42957409	1292
PA pH9_SPME	pH9	PA	2-Undecanone	25.114	52172308	1292
PA_SPMEpH7	pH7	PA	2-Undecanone	25.139	1.44E+08	1293
TSB pH5_SPME	pH5	TSB	indole	25.134	4376027	1292
TSB_SPMEpH7	pH7	TSB	indole	25.105	7933119	1292
5 mix pH3_SPME	pH3	5 mix	indole	25.206	3.18E+08	1295
KO pH5_SPME	pH5	KO	indole	25.244	4.57E+08	1296
KO1_SPMEpH7	pH7	KO	indole	25.215	5.77E+08	1295
TSB pH9_SPME	pH9	TSB	indole	25.156	3540314	1293
5 mix pH12_SPME	pH12	5 mix	indole	25.304	6.3E+08	1297
5 mix pH5_SPME	pH5	5 mix	indole	25.253	4.97E+08	1296
5 mix pH7_SPME	pH7	5 mix	indole	25.316	6.27E+08	1298
5 mix pH9_SPME	pH9	5 mix	indole	25.312	6.32E+08	1298

KO pH12_SPME	pH12	KO	indole	25.303	6.35E+08	1297
KO pH3_SPME	pH3	KO	indole	25.253	4.39E+08	1296
KO pH9_SPME	pH9	KO	indole	25.274	4.82E+08	1296
CF pH5_SPME	pH5	CF	2-Undecanol	25.438	20453512	1301
EA pH12_SPME	pH12	EA	Pyrazine, 2,5-dimethyl-3-(3-methylbutyl)-	25.769	5744332	1311
EA pH5_SPME	pH5	EA	Pyrazine, 2,5-dimethyl-3-(3-methylbutyl)-	25.769	2836312	1311
EA pH9_SPME	pH9	EA	Pyrazine, 2,5-dimethyl-3-(3-methylbutyl)-	25.775	3303877	1311
EA_SPMEpH7	pH7	EA	Pyrazine, 2,5-dimethyl-3-(3-methylbutyl)-	25.771	4795399	1311
EC pH3_SPME	pH3	EC	Pyrazine, 2,5-dimethyl-3-(3-methylbutyl)-	25.781	2073994	1311
PA pH9_SPME	pH9	PA	Pyrazine, 2,5-dimethyl-3-(3-methylbutyl)-	25.778	5211908	1311
PA_SPMEpH7	pH7	PA	Pyrazine, 2,5-dimethyl-3-(3-methylbutyl)-	25.769	6746967	1311
TSB pH12_SPME	pH12	TSB	Pyrazine, 2,5-dimethyl-3-(3-methylbutyl)-	25.769	6346049	1311
TSB pH5_SPME	pH5	TSB	Pyrazine, 2,5-dimethyl-3-(3-methylbutyl)-	25.773	2625122	1311
TSB pH9_SPME	pH9	TSB	Pyrazine, 2,5-dimethyl-3-(3-methylbutyl)-	25.777	5026316	1311
TSB_SPMEpH7	pH7	TSB	Pyrazine, 2,5-dimethyl-3-(3-methylbutyl)-	25.769	6435862	1311
TSB pH12_SPME	pH12	TSB	Pentadecane	26.088	2816605	1320
EC pH3_SPME	pH3	EC	Propanoic acid, 2-phenylethyl ester	27.062	2778536	1349
CF pH9_SPME	pH9	CF	Cyclopentasiloxane, decamethyl-	27.372	3745849	1359
EA pH12_SPME	pH12	EA	2-Dodecanone	27.52	4450129	1363
EA pH5_SPME	pH5	EA	2-Dodecanone	27.52	5582535	1363
EA pH9_SPME	pH9	EA	2-Dodecanone	27.522	4137008	1363
EA_SPMEpH7	pH7	EA	2-Dodecanone	27.519	4427005	1363
TSB pH12_SPME	pH12	TSB	3-methylbutyl 2-phenylethylidene amine	28.247	3134119	1385
CF pH5_SPME	pH5	CF	2-Dodecanone	28.509	10903870	1392
TSB pH3_SPME	pH3	TSB	siloxane	30.877	2113101	1466
EC pH3_SPME	pH3	EC	5-Decen-1-ol, acetate, (E)-	30.965	4831762	1469
EC pH7_SPME	pH7	EC	5-Decen-1-ol, acetate, (E)-	30.958	6719235	1469
EC pH9_SPME	pH9	EC	5-Decen-1-ol, acetate, (E)-	30.958	4869497	1469
PA pH3_SPME really	pH3	PA	5-Decen-1-ol, acetate, (E)-	30.96	4304308	1469
PA pH9_SPME	pH9	PA	5-Decen-1-ol, acetate, (E)-	30.962	7357237	1469
PA_SPMEpH7	pH7	PA	5-Decen-1-ol, acetate, (E)-	30.953	14440250	1469
EC pH5_SPME	pH5	EC	2-tridecanone	31.744	15675779	1494
EC pH9_SPME	pH9	EC	2-tridecanone	31.748	7727791	1494
PA pH12_SPME	pH12	PA	2-tridecanone	31.744	20819352	1494
5 mix pH5_SPME	pH5	5 mix	2-tridecanone	31.753	10030096	1494
CF pH5_SPME	pH5	CF	2-tridecanone	31.773	1.17E+08	1495
Cf_SPMEpH7	pH7	CF	2-tridecanone	31.751	6213450	1494
EC pH3_SPME	pH3	EC	2-tridecanone	31.756	6964244	1494

EC pH7_SPME	pH7	EC	2-tridecanone	31.752	9250689	1494
KO pH5_SPME	pH5	KO	2-tridecanone	31.752	19400777	1494
KO1_SPMEpH7	pH7	KO	2-tridecanone	31.753	10952173	1494
PA pH3_SPME really	pH3	PA	2-tridecanone	31.752	17698063	1494
PA pH9_SPME	pH9	PA	2-tridecanone	31.757	33831536	1494
PA_SPMEpH7	pH7	PA	2-tridecanone	31.757	58220070	1494
CF pH5_SPME	pH5	CF	2-Tridecanol	32.018	23300582	1502
CF pH9_SPME	pH9	CF	2-Tridecanol	32.56	3114266	1520
TSB pH3_SPME	pH3	TSB	2,2,4-Trimethyl-1,3-pentanediol diisobutyrate	34.554	8811849	1587
CF pH3_SPME	pH3	CF	Hexadecane	34.949	23647309	1600
KO pH3_SPME	pH3	KO	Hexadecane	34.958	37260809	1600
PA pH3_SPME really	pH3	PA	Hexadecane	34.953	31975249	1600
TSB pH3_SPME	pH3	TSB	Hexadecane	34.954	7434586	1600
CF pH3_SPME	pH3	CF	Octadecane	40.531	7540138	1800
EA pH3_SPME	pH3	EA	Octadecane	40.538	4130394	1800
EC pH3_SPME	pH3	EC	Octadecane	40.543	2564872	1800
KO pH3_SPME	pH3	KO	Octadecane	40.536	21707993	1800
PA pH3_SPME really	pH3	PA	Octadecane	40.535	12686926	1800
TSB pH3_SPME	pH3	TSB	Octadecane	40.54	4899658	1800

Table S3. Mean relative proportions (peak area) of microbial volatile organic compounds (MVOCs) identified for each bacterium grown on tryptone soya broth (TSB) and inoculated on three types of fruits; apple, kiwifruit, and orange. Volatiles were collected via Tenax® for 24 h, 3 days, and 5 days, and thermally desorbed. (CF: *Citrobacter freundii*; EC: *Enterobacter cloacae*; KO: *Klebsiella oxytoca*; PA: *Enterobacter (syn. Pantoea) agglomerans* and TSB media).

Sample_ID	Fruit	Technique	Active for Analysis	Type	RT [min]	Area	S
Kiwi_CF_3d_TEN1	Kiwi	Tenax	Ethyl butyrate	PEAK	5.319	21468113	
Kiwi_CF_3d_TEN1	Kiwi	Tenax	Indole	PEAK	22.506	7491340	
Kiwi_CF_3d_TEN1	Kiwi	Tenax	Ethyl hexanoate	PEAK	11.887	3290801	
Kiwi_CF_3d_TEN1	Kiwi	Tenax	Nonanal	PEAK	15.879	1113274	
Kiwi_CF_3d_TEN2	Kiwi	Tenax	Ethyl butyrate	PEAK	5.304	14678174	
Kiwi_CF_3d_TEN2	Kiwi	Tenax	Ethyl hexanoate	PEAK	11.895	2305086	
Kiwi_CF_3d_TEN2	Kiwi	Tenax	Nonanal	PEAK	15.877	963709	
Kiwi_CF_3d_TEN2	Kiwi	Tenax	Indole	PEAK	22.498	272649	
Kiwi_CF_3d_TEN3	Kiwi	Tenax	Ethyl butyrate	PEAK	5.324	17782137	
Kiwi_CF_3d_TEN3	Kiwi	Tenax	Indole	PEAK	22.504	2103888	

Kiwi_CF_3d_TEN3	Kiwi	Tenax	Ethyl hexanoate	PEAK	11.897	691633
Kiwi_CF_3d_TEN3	Kiwi	Tenax	Nonanal	PEAK	15.88	390511
Kiwi_CF_3d_TEN3	Kiwi	Tenax	D-limonene	PEAK	12.986	260725
Kiwi_CF_3d_TEN_control	Kiwi	Tenax	Ethyl butyrate	PEAK	5.316	19303438
Kiwi_CF_3d_TEN_control	Kiwi	Tenax	Ethyl hexanoate	PEAK	11.901	1043371
Kiwi_CF_3d_TEN_control	Kiwi	Tenax	Hexane, 2-4-dimethyl	PEAK	8.314	445596
Kiwi_CF_3d_TEN_control	Kiwi	Tenax	D-limonene	PEAK	12.999	308711
Kiwi_EC_3d_TEN1	Kiwi	Tenax	Ethyl butyrate	PEAK	5.285	11851484
Kiwi_EC_3d_TEN1	Kiwi	Tenax	Ethyl hexanoate	PEAK	11.878	1619369
Kiwi_EC_3d_TEN1	Kiwi	Tenax	Methyleugenol	PEAK	26.184	983142
Kiwi_EC_3d_TEN1	Kiwi	Tenax	D-limonene	PEAK	12.971	903540
Kiwi_EC_3d_TEN1	Kiwi	Tenax	Indole	PEAK	22.489	840049
Kiwi_EC_3d_TEN1	Kiwi	Tenax	Nonanal	PEAK	15.865	801761
Kiwi_EC_3d_TEN1	Kiwi	Tenax	Hexane, 2-4-dimethyl	PEAK	8.291	338586
Kiwi_EC_3d_TEN2	Kiwi	Tenax	Ethyl butyrate	PEAK	5.315	23463342
Kiwi_EC_3d_TEN2	Kiwi	Tenax	Ethyl hexanoate	PEAK	11.884	3887461
Kiwi_EC_3d_TEN2	Kiwi	Tenax	Nonanal	PEAK	15.874	1939086
Kiwi_EC_3d_TEN2	Kiwi	Tenax	D-limonene	PEAK	12.978	1597352
Kiwi_EC_3d_TEN2	Kiwi	Tenax	Methyleugenol	PEAK	26.187	899028
Kiwi_EC_3d_TEN2	Kiwi	Tenax	Hexane, 2-4-dimethyl	PEAK	8.3	443965
Kiwi_EC_3d_TEN3	Kiwi	Tenax	Ethyl butyrate	PEAK	5.316	44861770
Kiwi_EC_3d_TEN3	Kiwi	Tenax	Ethyl hexanoate	PEAK	11.892	10961118
Kiwi_EC_3d_TEN3	Kiwi	Tenax	Methyleugenol	PEAK	26.189	2047541
Kiwi_EC_3d_TEN3	Kiwi	Tenax	Nonanal	PEAK	15.877	728417
Kiwi_EC_3d_TEN3	Kiwi	Tenax	Indole	PEAK	22.5	669560
Kiwi_EC_3d_TEN3	Kiwi	Tenax	Hexane, 2-4-dimethyl	PEAK	8.305	612848
Kiwi_EC_3d_TEN3	Kiwi	Tenax	D-limonene	PEAK	12.986	542614
Kiwi_EC_3d_TEN_control	Kiwi	Tenax	Ethyl butyrate	PEAK	5.313	11374451
Kiwi_EC_3d_TEN_control	Kiwi	Tenax	Ethyl hexanoate	PEAK	11.894	1928004
Kiwi_EC_3d_TEN_control	Kiwi	Tenax	Indole	PEAK	22.491	786967
Kiwi_EC_3d_TEN_control	Kiwi	Tenax	Hexane, 2-4-dimethyl	PEAK	8.3	441324
Apple_EC_24h_SPME1	Apple	SPME	butyl acetate	PEAK	5.694	14225923
Apple_EC_24h_SPME1	Apple	SPME	Ethyl octanoate	PEAK	19.103	2762826
Apple_EC_24h_SPME1	Apple	SPME	α-farnesene	PEAK	29.476	625924
Apple_EC_24h_SPME1	Apple	SPME	Methyleugenol	PEAK	26.246	276817
Apple_EC_24h_SPME2	Apple	SPME	butyl acetate	PEAK	5.72	3343417
Apple_EC_24h_SPME2	Apple	SPME	Indole	PEAK	22.58	2376810
Apple_EC_24h_SPME2	Apple	SPME	Ethyl butyrate	PEAK	5.158	1379852
Apple_EC_24h_SPME2	Apple	SPME	Ethyl octanoate	PEAK	19.344	1178735
Apple_EC_24h_SPME2	Apple	SPME	α-farnesene	PEAK	29.407	804150
Apple_EC_24h_SPME_control	Apple	SPME	butyl acetate	PEAK	5.696	6668102
Apple_EC_24h_SPME_control	Apple	SPME	α-farnesene	PEAK	29.483	2588618
Apple_EC_24h_SPME_control	Apple	SPME	Indole	PEAK	22.579	1614458
Apple_EC_24h_SPME_control	Apple	SPME	hexyl hexanoate	PEAK	25.767	950521
Kiwi_EC_24h_SPME1	Kiwi	SPME	Indole	PEAK	22.497	5875359
Kiwi_EC_24h_SPME1	Kiwi	SPME	Nonanal	PEAK	15.854	514310

Kiwi_EC_24h_SPME1	Kiwi	SPME	D-limonene	PEAK	12.978	389740
Kiwi_EC_24h_SPME1	Kiwi	SPME	Methyleugenol	PEAK	26.162	306745
Kiwi_EC_24h_SPME1	Kiwi	SPME	Ethyl butyrate	PEAK	5.277	192051
Kiwi_EC_24h_SPME2	Kiwi	SPME	Ethyl butyrate	PEAK	5.271	23607726
Kiwi_EC_24h_SPME2	Kiwi	SPME	Pyrazine,2,5-dimethyl	PEAK	8.809	1183517
Kiwi_EC_24h_SPME2	Kiwi	SPME	Indole	PEAK	22.474	884801
Kiwi_EC_24h_SPME2	Kiwi	SPME	Nonanal	PEAK	15.845	457435
Kiwi_EC_24h_SPME2	Kiwi	SPME	D-limonene	PEAK	12.974	416295
Kiwi_EC_24h_SPME3	Kiwi	SPME	D-limonene	PEAK	12.967	5768377
Kiwi_EC_24h_SPME3	Kiwi	SPME	Indole	PEAK	22.501	3100319
Kiwi_EC_24h_SPME3	Kiwi	SPME	Nonanal	PEAK	15.858	1363873
Kiwi_EC_24h_SPME3	Kiwi	SPME	butyl acetate	PEAK	5.641	1009640
Kiwi_EC_24h_SPME3	Kiwi	SPME	Ethyl butyrate	PEAK	5.286	553972
Kiwi_EC_24h_SPME3	Kiwi	SPME	Phenylethyl alcohol	PEAK	16.143	460102
Kiwi_EC_24h_SPME3	Kiwi	SPME	Methyleugenol	PEAK	26.168	218207
Kiwi_EC_24h_SPME_control	Kiwi	SPME	Indole	PEAK	22.508	10712965
Kiwi_EC_24h_SPME_control	Kiwi	SPME	Nonanal	PEAK	15.858	378308
Orange_EC_24h_SMPE1	Orange	SPME	D-limonene	PEAK	13.139	594458999
Orange_EC_24h_SMPE1	Orange	SPME	Ethyl octanoate	PEAK	19.262	42224382
Orange_EC_24h_SMPE1	Orange	SPME	β-pinene	PEAK	11.545	20894613
Orange_EC_24h_SMPE1	Orange	SPME	Methyl octanoate	PEAK	16.585	6571971
Orange_EC_24h_SMPE1	Orange	SPME	Indole	PEAK	22.511	5184060
Orange_EC_24h_SMPE1	Orange	SPME	Naphtalene	PEAK	28.798	3750840
Orange_EC_24h_SMPE1	Orange	SPME	Ethyl butyrate	PEAK	5.306	3512001
Orange_EC_24h_SMPE1	Orange	SPME	Butyl hexanoate	PEAK	19.073	3299956
Orange_EC_24h_SMPE1	Orange	SPME	Eugenol	PEAK	24.551	3185061
Orange_EC_24h_SMPE1	Orange	SPME	Ethyl hexanoate	PEAK	13.702	3047946
Orange_EC_24h_SMPE1	Orange	SPME	Caryophyllene	PEAK	26.776	2768208
Orange_EC_24h_SMPE1	Orange	SPME	Methyleugenol	PEAK	26.152	1873947
Orange_EC_24h_SMPE3	Orange	SPME	Ethyl octanoate	PEAK	19.256	53415661
Orange_EC_24h_SMPE3	Orange	SPME	β-pinene	PEAK	11.543	21997696
Orange_EC_24h_SMPE3	Orange	SPME	Phenylethyl alcohol	PEAK	16.186	7205348
Orange_EC_24h_SMPE3	Orange	SPME	Hexyl hexanoate	PEAK	25.799	7190221
Orange_EC_24h_SMPE3	Orange	SPME	Ethyl hexanoate	PEAK	13.682	6000077
Orange_EC_24h_SMPE3	Orange	SPME	Ethyl butyrate	PEAK	5.305	5369578
Orange_EC_24h_SMPE3	Orange	SPME	Methyl octanoate	PEAK	16.573	4747304
Orange_EC_24h_SMPE3	Orange	SPME	Naphtalene	PEAK	28.79	4641252
Orange_EC_24h_SMPE3	Orange	SPME	Eugenol	PEAK	24.536	1915581
Orange_EC_24h_SMPE3	Orange	SPME	Caryophyllene	PEAK	26.764	1744909
Orange_EC_24h_SMPE3	Orange	SPME	Methyleugenol	PEAK	26.136	1516072
Orange_EC_24h_SMPE2	Orange	SPME	D-limonene	PEAK	13.181	914971786
Orange_EC_24h_SMPE2	Orange	SPME	Ethyl octanoate	PEAK	19.277	89428675
Orange_EC_24h_SMPE2	Orange	SPME	β-pinene	PEAK	11.547	35604819
Orange_EC_24h_SMPE2	Orange	SPME	Ethyl butyrate	PEAK	5.298	17297485

Orange_EC_24h_SMPE2	Orange	SPME	Phenylethyl alcohol	PEAK	16.185	13134726
Orange_EC_24h_SMPE2	Orange	SPME	Methyl octanoate	PEAK	16.578	11772745
Orange_EC_24h_SMPE2	Orange	SPME	Ethyl hexanoate	PEAK	13.692	8705033
Orange_EC_24h_SMPE2	Orange	SPME	Indole	PEAK	22.507	7462988
Orange_EC_24h_SMPE2	Orange	SPME	Naphtalene	PEAK	28.791	3959155
Orange_EC_24h_SMPE2	Orange	SPME	Caryophyllene	PEAK	26.764	1978159
Orange_EC_24h_SMPE2	Orange	SPME	Eugenol	PEAK	24.54	1947880
Orange_EC_24h_SMPE2	Orange	SPME	Methyleugenol	PEAK	26.14	905220
Orange_EC_24h_SPME_control	Orange	SPME	D-limonene	PEAK	13.19	972661360
Orange_EC_24h_SPME_control	Orange	SPME	Ethyl octanoate	PEAK	19.274	77153293
Orange_EC_24h_SPME_control	Orange	SPME	Ethyl hexanoate	PEAK	13.704	38263565
Orange_EC_24h_SPME_control	Orange	SPME	Phenylethyl alcohol	PEAK	16.195	37971098
Orange_EC_24h_SPME_control	Orange	SPME	β-pinene	PEAK	11.541	34965957
Orange_EC_24h_SPME_control	Orange	SPME	Ethyl butyrate	PEAK	5.283	25015838
Orange_EC_24h_SPME_control	Orange	SPME	Methyl octanoate	PEAK	16.584	9371416
Orange_EC_24h_SPME_control	Orange	SPME	Naphtalene	PEAK	28.795	4958250
Orange_EC_24h_SPME_control	Orange	SPME	Caryophyllene	PEAK	26.764	4490428
Orange_EC_24h_SPME_control	Orange	SPME	Indole	PEAK	22.507	1860460
Kiwi_EC_72h_SPMEpool	Kiwi	SPME	Ethyl butyrate	PEAK	5.312	39284086
Kiwi_EC_72h_SPMEpool	Kiwi	SPME	Nonanal	PEAK	15.845	2383420
Kiwi_EC_72h_SPMEpool	Kiwi	SPME	Methyleugenol	PEAK	26.141	2370041
Kiwi_EC_72h_SPMEpool	Kiwi	SPME	D-limonene	PEAK	12.961	2171550
Kiwi_CF_72h_SPMEpool	Kiwi	SPME	Ethyl butyrate	PEAK	5.295	15583096
Kiwi_CF_72h_SPMEpool	Kiwi	SPME	Indole	PEAK	22.463	3374463
Kiwi_CF_72h_SPMEpool	Kiwi	SPME	Nonanal	PEAK	15.843	1126580
Kiwi_CF_72h_SPMEpool	Kiwi	SPME	Phenylethyl alcohol	PEAK	16.046	1057600
Kiwi_CF_72h_SPMEpool	Kiwi	SPME	Methyleugenol	PEAK	26.14	527918
Kiwi_CF_72h_SPMEpool	Kiwi	SPME	Naphtalene	PEAK	28.688	152204
Kiwi_KO_24h_SPME1	Kiwi	SPME	D-limonene	PEAK	13.057	78124462
Kiwi_KO_24h_SPME2	Kiwi	SPME	Indole	PEAK	22.546	5943823
Kiwi_KO_24h_SPME2	Kiwi	SPME	D-limonene	PEAK	13.059	2219058
Kiwi_KO_24h_SPME2	Kiwi	SPME	Phenylethyl alcohol	PEAK	16.136	299174
Kiwi_KO_24h_SPME3	Kiwi	SPME	D-limonene	PEAK	13.04	9502109
Kiwi_KO_24h_SPME3	Kiwi	SPME	Indole	PEAK	22.586	1284036
Kiwi_KO_24h_SPME3	Kiwi	SPME	hexyl hexanoate	PEAK	25.838	303757
Kiwi_KO_24h_SPME3	Kiwi	SPME	β-pinene	PEAK	11.564	222461
Kiwi_KO_24h_SPME3	Kiwi	SPME	Butyl hexanoate	PEAK	19.105	214792
Kiwi_KO_24h_SPME_control	Kiwi	SPME	D-limonene	PEAK	13.039	8341676
Kiwi_KO_24h_SPME_control	Kiwi	SPME	Indole	PEAK	22.578	1016243
Kiwi_KO_24h_SPME_control	Kiwi	SPME	Ethyl octanoate	PEAK	19.267	401570
Kiwi_KO_24h_SPME_control	Kiwi	SPME	Butyl hexanoate	PEAK	19.096	167606
Orange_KO_24h_SPME1	Orange	SPME	D-limonene	PEAK	13.184	1023503089
Orange_KO_24h_SPME1	Orange	SPME	Ethyl octanoate	PEAK	19.278	92618280

Orange_KO_24h_SPME1	Orange	SPME	Hexyl hexanoate	PEAK	25.816	55534464
Orange_KO_24h_SPME1	Orange	SPME	β-pinene	PEAK	11.556	37629836
Orange_KO_24h_SPME1	Orange	SPME	Butyl hexanoate	PEAK	19.077	33609205
Orange_KO_24h_SPME1	Orange	SPME	Ethyl hexanoate	PEAK	13.694	21529855
Orange_KO_24h_SPME1	Orange	SPME	Naphtalene	PEAK	28.805	17437196
Orange_KO_24h_SPME1	Orange	SPME	Phenylethyl alcohol	PEAK	16.186	12409314
Orange_KO_24h_SPME1	Orange	SPME	Ethyl butyrate	PEAK	5.299	8730857
Orange_KO_24h_SPME1	Orange	SPME	Methyl octanoate	PEAK	16.587	8602502
Orange_KO_24h_SPME1	Orange	SPME	Caryophyllene	PEAK	26.784	6177041
Orange_KO_24h_SPME1	Orange	SPME	Indole	PEAK	22.52	3663370
Orange_KO_24h_SPME2	Orange	SPME	Ethyl butyrate	PEAK	5.314	10916416
Orange_KO_24h_SPME2	Orange	SPME	β-pinene	PEAK	11.55	27835992
Orange_KO_24h_SPME2	Orange	SPME	D-limonene	PEAK	13.176	945949859
Orange_KO_24h_SPME2	Orange	SPME	Ethyl hexanoate	PEAK	13.699	16908097
Orange_KO_24h_SPME2	Orange	SPME	Phenylethyl alcohol	PEAK	16.19	8556901
Orange_KO_24h_SPME2	Orange	SPME	Methyl octanoate	PEAK	16.584	5614138
Orange_KO_24h_SPME2	Orange	SPME	Butyl hexanoate	PEAK	19.068	30580242
Orange_KO_24h_SPME2	Orange	SPME	Ethyl octanoate	PEAK	19.267	54190715
Orange_KO_24h_SPME2	Orange	SPME	Hexyl hexanoate	PEAK	25.709	28167526
Orange_KO_24h_SPME2	Orange	SPME	Caryophyllene	PEAK	26.775	3477822
Orange_KO_24h_SPME2	Orange	SPME	Naphtalene	PEAK	28.797	7700674
Orange_KO_24h_SPME3	Orange	SPME	Ethyl butyrate	PEAK	5.296	2094112
Orange_KO_24h_SPME3	Orange	SPME	β-pinene	PEAK	11.542	5256301
Orange_KO_24h_SPME3	Orange	SPME	D-limonene	PEAK	13.065	255272015
Orange_KO_24h_SPME3	Orange	SPME	Ethyl hexanoate	PEAK	13.683	6178771
Orange_KO_24h_SPME3	Orange	SPME	Phenylethyl alcohol	PEAK	16.185	2585071
Orange_KO_24h_SPME3	Orange	SPME	Methyl octanoate	PEAK	16.582	1937601
Orange_KO_24h_SPME3	Orange	SPME	Butyl hexanoate	PEAK	19.058	10073886
Orange_KO_24h_SPME3	Orange	SPME	Ethyl octanoate	PEAK	19.245	16847337
Orange_KO_24h_SPME3	Orange	SPME	Hexyl hexanoate	PEAK	25.708	16593567
Orange_KO_24h_SPME3	Orange	SPME	Caryophyllene	PEAK	26.776	2051444
Orange_KO_24h_SPME3	Orange	SPME	Naphtalene	PEAK	28.789	4076883
Orange_KO_24h_SPME_control	Orange	SPME	Ethyl butyrate	PEAK	5.308	1778822
Orange_KO_24h_SPME_control	Orange	SPME	β-pinene	PEAK	11.538	5314276
Orange_KO_24h_SPME_control	Orange	SPME	D-limonene	PEAK	13.071	252798769
Orange_KO_24h_SPME_control	Orange	SPME	Ethyl hexanoate	PEAK	13.682	3180870
Orange_KO_24h_SPME_control	Orange	SPME	Methyl octanoate	PEAK	16.573	1352555
Orange_KO_24h_SPME_control	Orange	SPME	Butyl hexanoate	PEAK	19.06	5665957
Orange_KO_24h_SPME_control	Orange	SPME	Ethyl octanoate	PEAK	19.237	18461273
Orange_KO_24h_SPME_control	Orange	SPME	Indole	PEAK	22.521	1278371
Orange_KO_24h_SPME_control	Orange	SPME	Eugenol	PEAK	24.538	485614
Orange_KO_24h_SPME_control	Orange	SPME	Hexyl hexanoate	PEAK	25.702	3941211
Orange_KO_24h_SPME_control	Orange	SPME	Methyleugenol	PEAK	26.191	293107
Orange_KO_24h_SPME_control	Orange	SPME	Caryophyllene	PEAK	26.774	709843

Orange_KO_24h_SPME_control	Orange	SPME	Naphtalene	PEAK	28.794	1198970

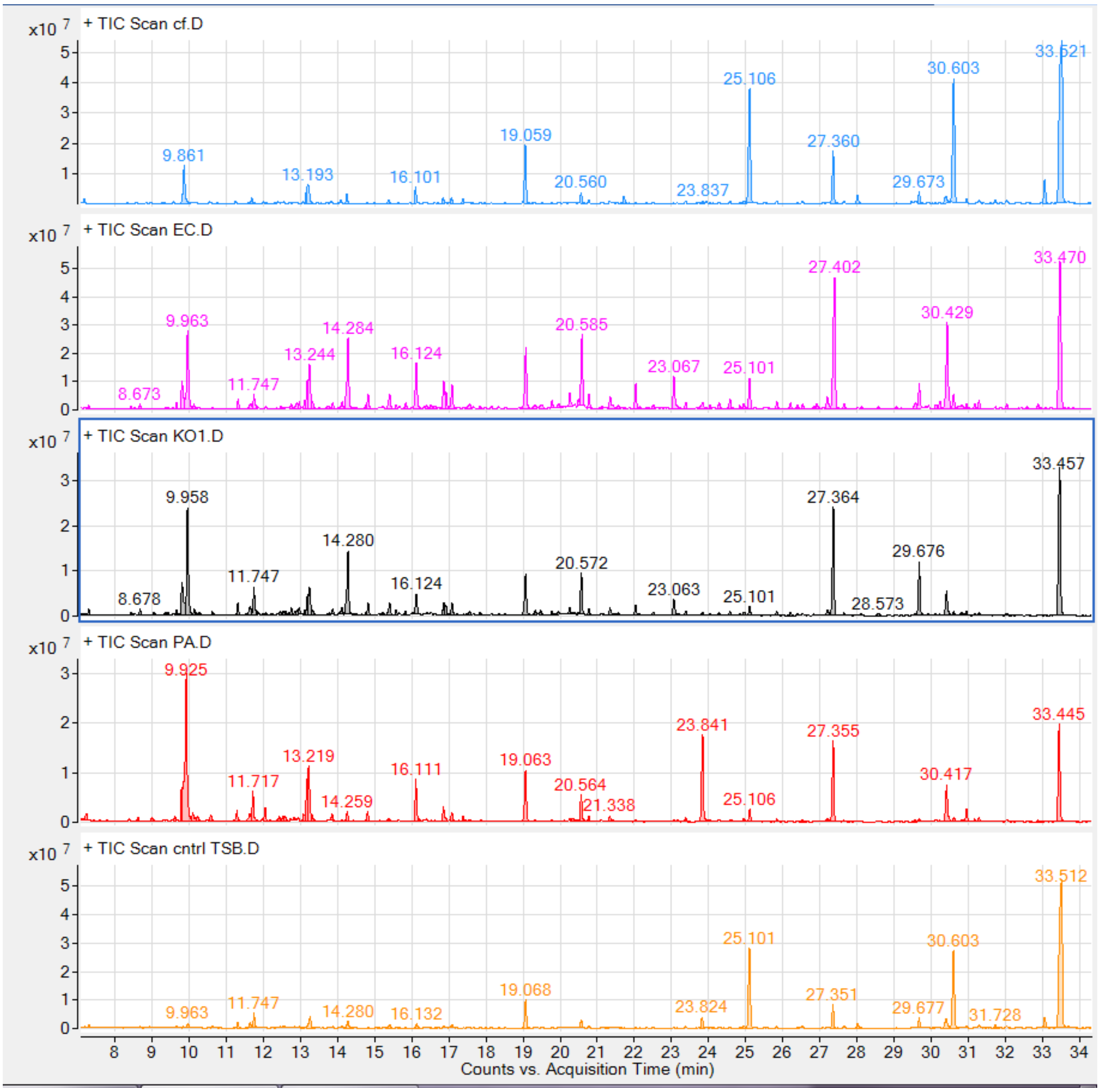

Figure S1. Representative chromatograph profiles of four bacterial species grown on tryptone soya broth (TSB), collected on Tenax® for 24 h and solvent-extracted. From top to bottom: CF-*Citrobacter freundii*; EC-*Enterobacter cloacae*; KO-*Klebsiella oxytoca*; PA- Enterobacter *(sy. Pantoea) agglomerans* and TSB media.

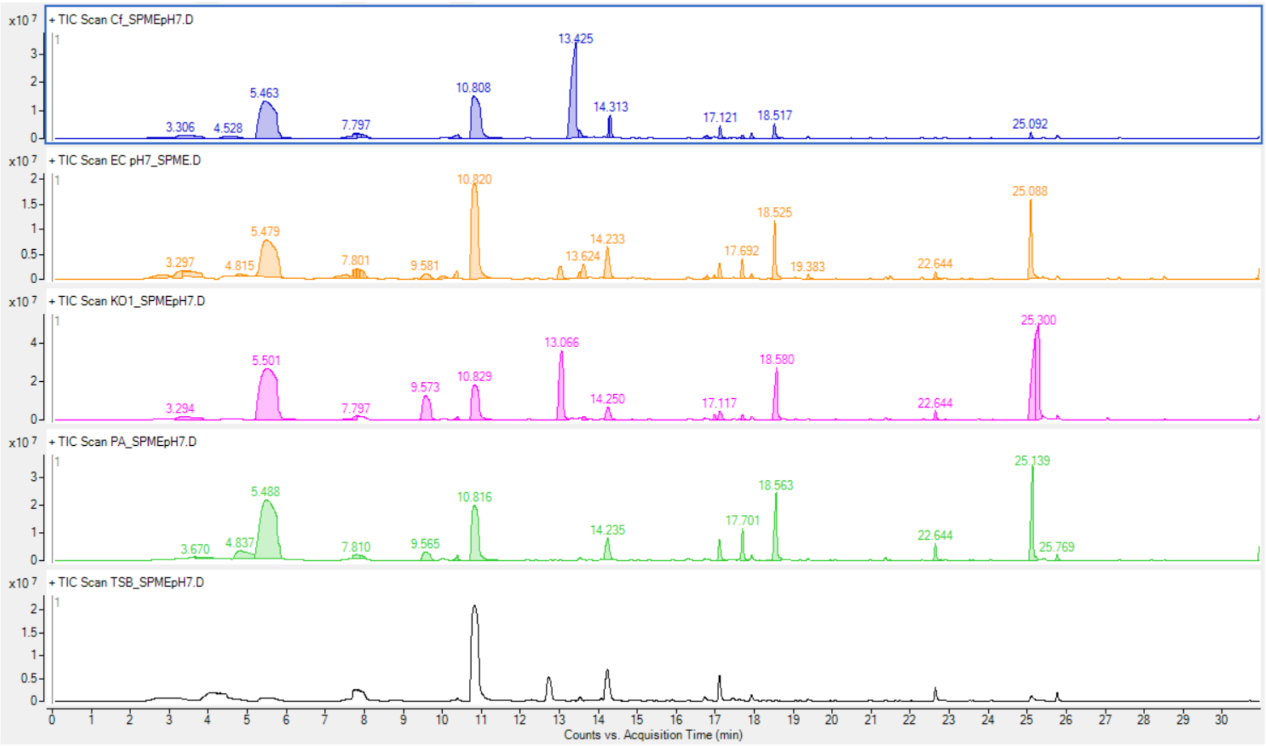

Figure S2. Representative chromatograph profiles of four bacterial species grown on tryptone soya broth (TSB), collected with solid phase micro-extraction (SPME) fibre for 1 h and thermally desorbed. From top to bottom: CF-*Citrobacter freundii*; EC-*Enterobacter cloacae*; KO-*Klebsiella oxytoca*; PA- *Enterobacter (syn. Pantoea) agglomerans* and TSB media.

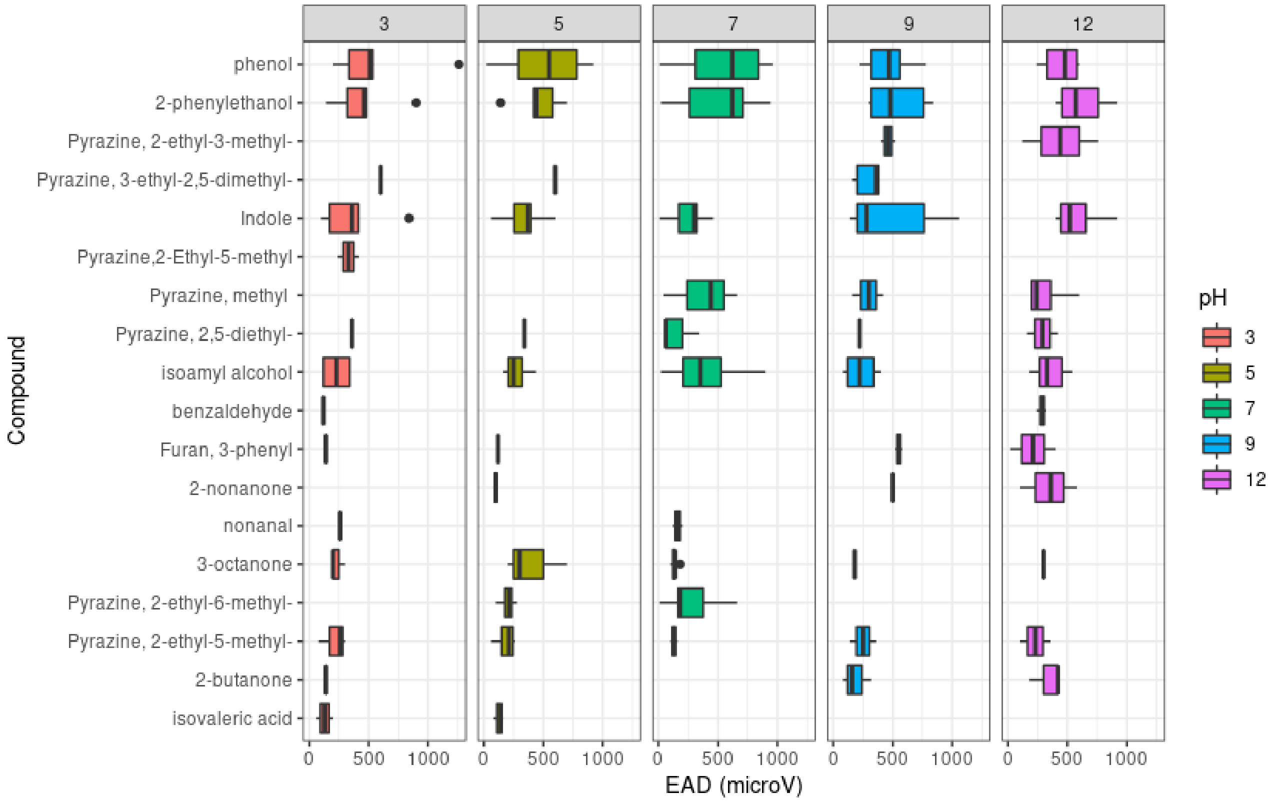

Figure S3. Olfactory responses from solid phase micro-extraction (SPME) - Gas-chromatograph coupled with electroantennographic detection (SPME-GC/EAD) of virgin females to microbial volatile organic compounds produced by a mix of four Enterobactericeae bacteria adjusted to pH 3,5,7,9 and 12.
